# Normalization of oligonucleotide arrays based on the least-variant set of genes

**DOI:** 10.1186/1471-2105-9-140

**Published:** 2008-03-05

**Authors:** Stefano Calza, Davide Valentini, Yudi Pawitan

**Affiliations:** 1 Department of Medical Epidemiology and Biostatistics, Karolinska Institutet, Stockholm, Sweden; 2 Department of Biomedical Sciences and Biotechnology, University of Brescia, Italy

## Abstract

**Background:**

It is well known that the normalization step of microarray data makes a difference in the downstream analysis. All normalization methods rely on certain assumptions, so differences in results can be traced to different sensitivities to violation of the assumptions. Illustrating the lack of robustness, in a striking spike-in experiment all existing normalization methods fail because of an imbalance between up- and down-regulated genes. This means it is still important to develop a normalization method that is robust against violation of the standard assumptions

**Results:**

We develop a new algorithm based on identification of the least-variant set (LVS) of genes across the arrays. The array-to-array variation is evaluated in the robust linear model fit of pre-normalized probe-level data. The genes are then used as a reference set for a non-linear normalization. The method is applicable to any existing expression summaries, such as MAS5 or RMA.

**Conclusion:**

We show that LVS normalization outperforms other normalization methods when the standard assumptions are not satisfied. In the complex spike-in study, LVS performs similarly to the ideal (in practice unknown) housekeeping-gene normalization. An R package called lvs is available in .

## Background

High-throughput microarray technologies are becoming the norm in genetic and molecular research. Nevertheless, some steps in the preprocessing of the data prior to main analyses still remain problematic, as there is no universally accepted procedure for background correction, expression-value summarization and normalization. Here we are focusing on the normalization step of Affymetrix expression arrays, whose main purpose is to remove any systematic non-biological array-to-array variation. It is well known that (i) a noisy technical variation exists between arrays [1] due to many factors such as mRNA quantities, scanner settings, instrument operator, etc., and (ii) the choice of normalization method can make a substantial impact to the final results [2].

Currently, the quantile normalization [3,4], global normalization [5] and loess normalization [6] are among the most commonly used. However, all these methods rely on sensitive assumptions that may be violated in real applications. To illustrate the impact of normalization step on final results, Table 1 reports the percentages of concordance in the number of genes declared differentially expressed (DE) between different normalization procedure applied to the same expression measure (MAS5). The gene expression measurements were taken from of skeletal muscle biopsies from 12 Duchenne muscular dystrophy (DMD) patients and 11 unaffected control patients [7]. The same analysis method [8] was used for all the algorithms. For 0.1% false discovery rate limit, the number of DE genes varied from 97 to 134, and the concordance rate goes as low as 70%.

**Table 1:** Absolute and relative (in parentheses) concordances between different normalization algorithms applied to MAS5 expression values. The numbers of genes declared DE are determined using the local false discovery rate [8] at 0.1% limit. Percentages in parenthesis are relative to the method in the column headings. The last line reports the percentages of over-expressed genes among those declared DE.

	Global	Invariantset	Quantile	Loess
Global	132	110 (0.82)	93 (0.96)	100 (0.96)
Invariantset	110 (0.83)	134	97 (1.00)	103 (0.99)
Quantile	93 (0.70)	97 (0.72)	97	92 (0.88)
Loess	100 (0.76)	103 (0.77)	92 (0.95)	104

% over-expressed	0.88	0.93	0.9	0.93

All normalization methods need a reference set of genes that do not vary between samples. Most methods in fact use the whole set of genes as the reference set, and this choice is justified with two key assumptions [3,9] that (i) the great majority of genes do not vary between samples, and (ii) the distribution of up- and down-regulated genes is approximatively symmetric. Under these assumptions, the simple global-mean normalization, for example, involves making all arrays have the same mean. The methods are not robust against violation of these assumptions: when either of the two assumptions is not satisfied, existing normalization procedures are not trustworthy. The problem is that, in practice, these assumptions are rarely checked. Furthermore, it is not usually stated at what proportion the 'great majority' should be, but statistically we should probably expect at least 90%. Much smaller proportions than that would undermine the methods; for example, if 40% of the genes vary, it is no longer credible that the global mean should be constant across the arrays.

Spike-in experiments have been the key tool to establish current normalization schemes. Most of these experiments are typically quite simple, involving only a few spike-in genes. For these experiments, most existing normalization methods work well. However, for the so-called Golden-Spike data [10], almost 4000 out of 14,010 genes are spiked, among which 1,331 are differentially expressed (DE) and 2,535 are nonDE.

The Golden-Spike experiment is contrived, but it is important in revealing the lack of robustness of the existing normalization methods. Because all the DE genes are up-regulated, hence violating the balanced regulation assumption, all of the current normalization methods fails with this dataset. In many real studies, unbalanced regulation might reasonably happen [11-13]. This scenario has been already investigated in two-color microarrays [14] and raises some question marks over the existing procedures. A different sensitivity to violation of this assumption might explain differences in performance of the methods. Searching for a safer and more robust normalization procedure has been the motivation for this paper.

The so-called housekeeping genes, i.e. genes involved in the basic maintenance of the cells, might be considered a perfect reference set for normalization. In fact, they are used for normalization of PCR assays [15,16]. To survive, every cell is supposed to express them approximately at the same level [17], so we do not expect the expression of these genes to vary between samples. Affymetrix arrays contain a set of possible housekeeping genes, usually used for quality control procedures, but also suggested as the optimal reference for normalizing the arrays. Nevertheless, a broad body of evidence exits that genes traditionally considered as housekeeping genes are in reality not invariant under a range of experimental and pathological conditions [18-20]. Specific examples of the failure of normalization based on a priori housekeeping genes were given in [21].

A data-driven procedure for identifying genes that do not vary across samples, and therefore might be a good reference set for normalization, leads to the so-called 'invariant-set normalization' [22,23]. The procedure selects the set of genes to use as a reference for normalization in a pairwise fashion. This is done by selecting genes whose ranks are invariant between each sample and a reference distribution, e.g. a pseudo-median sample.

Our approach here is also based on data-driven housekeeping genes by identifying genes that vary the least between arrays. Instead of using pairwise comparison between samples, we exploit the total information from all the samples. The information is extracted from the probe-level data, by partitioning the observed variability into array-to-array variation, within-probeset variation and residual variation. Probesets whose array-to-array variability are below a given quantile are called the 'least-variant set' (LVS), and they provide the reference set for normalization.

To summarize our novel contribution, we have developed the LVS normalization and studied its performance in several spike-in experiments. We show that LVS normalization (i) performs similarly to other normalization methods when the standard assumptions are satisfied, but (ii) performs better when the standard assumptions are violated. In fact, in the latter case, LVS performs similarly to the ideal (in practice unknown) housekeeping-gene normalization. This means that the LVS normalization is robust against violation of the standard assumptions of normalization, so it is more widely and more safely applicable than the current normalization methods.

## Methods

The LVS normalization is based on a two-step procedure. The first step operates at the probe-level data in order to estimate the component of variance due to array-to-array variability. This step is a multi-array procedure, using all the arrays in order to identify genes that vary least between the arrays. If the number of samples is large, a proper subset should be used for faster computation. The second step involves a non-linear fit of the LVS genes from individual arrays against those from a reference array, such as a pseudo-median array.

### Identification of the LVS genes

To identify the LVS genes, we analyse the probe-level data. Each probeset may contain from 8 to 20 pairs of perfect match (PM) and mismatch (MM) probes. First, the PM data is corrected for background; in our examples we use the so-called ideal mismatch (IMM) [5], but in principle any background correction method may be used. In Figure S3, Additional file 1, we show that different methods would produce similar LVS genes. Then, for each gene, specify the model

(1) log_2_(PM_*ij*_) = *μ *+ *α*_*i *_+ *β*_*j *_+ *ε*_*ij*_,

where PM_*ij *_is the background-corrected PM value for the *j*th probe from the *i*th array, *i *= 1,...,*n *and *j *= 1,...,*J*. *μ *is the grand mean parameter, *α*_*i *_is the *i*th array effect, and *β*_*j *_is the *j*th probe effect. This log-linear model was the basis for the RMA summary measure [24]. It was similar to, but not the same with, the Li and Wong model [23], which uses a multiplicative term plus noise, so we cannot take log and get a log-linear model.

The model was fitted by a robust M-estimation method [25], already implemented by the R package affyPLM [26]. The array-to-array variability is captured by the *χ*^2 ^test statistic, computed by

(2) $$\chi^{2}={\hat{\alpha }}^{\prime }V^{-1}\hat{\alpha },$$

where $\hat{\alpha }$ is the vector of estimated *α*_*i*_'s, and *V *is its estimated covariance matrix. These quantities are available from the robust linear model fit.

The array effect *α*_*i *_includes both the technical artifact *t*_*i *_and real biological effect *b*_*i*_, so that

(3) *α*_*i *_= *t*_*i *_+ *b*_*i*_.

The ideal housekeeping genes are those with *b*_*i *_≡ 0 for all *i*, thus allowing for the estimation of the remaining systematic variation that comes solely from technical sources. The LVS genes are the data-based estimation of these housekeeping genes.

**Figure 1:**
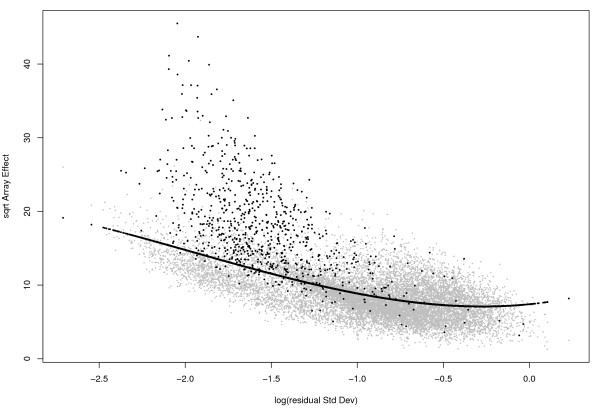
**RA-plot for Golden-Spike data**. This plot shows the array-to-array variablility vs residual variance from the probe level linear model. The black line is the quantile regression curve at proportion *τ *= 0.6. The black points correspond to genes with FC≥ 2.

Suppose for the moment that in model (3) *t*_*i *_and *b*_*i *_are independent random effects. Then, for genes with the same technical variance, the total array-to-array variability for housekeeping genes should be less than that for non-housekeeping genes. This means that when we compare the *χ*^2 ^statistics among the genes, those with smaller values are more likely to come from genes with *b*_*i *_≡ 0. Since the value of the statistic is determined by the residual variance, our assessment must also take it into account. The relationship between the *χ*^2 ^statistic for array effects and the residual standard deviation can be seen graphically (eg Figure 1), hereafter called the 'RA-plot'.

Thus, in practice, to determine the LVS genes, we fit a nonparametric quantile function [27,28] of the array *χ*^2 ^statistic (on the square root scale) as a function of the logarithm of residual standard deviation (SD), and declare those genes that fall under the curve as the LVS genes. In analogy with classical linear models where the conditional mean of the response variable is modelled as a function of some covariates, quantile regression aims at modelling any chosen quantile of the response variable a a function of the covariates. In our current application the quantile function is 'nonparametric' in the sense that it is not based on an explicit functional form, but on local smoothing of the data. We need to set a proportion *τ*, below which we expect genes not to vary between samples. In our experience *τ *= 60% is a reasonable choice, since we expect no more than 40% of the genes to be vary significantly between arrays. Nevertheless, it might be possible to conceive of an experimental situation were a higher proportion of genes are expected to be regulated, so the user needs to tune the value of *τ *accordingly.

This step works on multi-array basis, requiring all the arrays for the analysis. For a large number of arrays, memory requirement can be reduced by analysing limited number of probes at a time. Furthermore, to reduce computational burden, the analysis might also be performed on a random sub-sample of the data.

### Non-linear normalization on the LVS genes

Once the LVS genes are identified, the normalization algorithm works on the individual arrays by fitting a spline smoother between the arrays and an arbitrary reference array. The latter is, for example, a pseudo-median array or any user-specified array. The curve fitted through the least variant genes is then used to map intensities of all the genes in each array to be normalized.

This normalization step might be performed either after expression summarization or at the probe-level, prior to other preprocessing procedures. Any expression summary (e.g. MAS5-style, RMA-style, etc.) might be used. For all analyses in this paper, step 2 is applied after background correction and expression summarization. Finally, note that this step is single-array based, i.e., not requiring all the samples at the same time. This reduces the memory requirement during computation.

### Competing normalization procedures

The so-called global or constant normalization method is typically used by the Affymetrix Microarray Suite [5]. Each sample is rescaled to have mean set to some arbitrary target value (usually 500). This is achieved by dividing each sample values by a scaling factor obtained as the ratio between the target value and the sample mean. While the standard MAS5 algorithm works on the original scale, in our implementation we work on the log scale with zero target value, simply subtracting each sample by its mean.

While the global normalization assumes a linear relationship between the arrays, the invariant-set normalization [22,23] uses a non-linear regression to normalize data. A subset of genes is first selected based on comparing the ranks of the expression values in each sample to a reference array. The idea is that invariant genes, supposedly nonDE genes, should consistently have low ranks in each sample. A local regression is then fitted on the subset of invariant genes to get a normalization curve.

Quantile normalization [3,4] is a multichip procedure, were the expression distribution (across genes) of each sample is forced to be the same as the distribution of a reference sample. The reference might be any of the samples or any derived one, such as the pseudo-median sample.

## Datasets

Three freely-available data sets have been used to evaluate the proposed normalization procedure. All of these data sets are from spike-in experiments, i.e. produced by controlled experiments with known RNA intensities or predefined mutual relationships.

### Golden-Spike data

The so-called 'Golden-Spike' experiment for Affymetrix arrays designed by [10] provides a dataset of 3,860 RNA species, where 100–200 RNAs were spiked in at fold-change (FC) level ranging from 1.2 to 4-fold, while a set of 2,551 RNA species was spiked-in at a constant (FC = 1) level. Data were designed as a two-group comparison, spike-in (S) versus control (C) (n = 3 each), with overall 9.5% genes over-expressed in S versus C. Out of 14,010 probesets on this DrosGenome1 chip, 1,331 had FC>1, among which 650 had FC>2, 2,535 had FC = 1 and 10,144 were 'empty'. The FC1 genes are thus the ideal housekeeping genes, and provide the perfect reference set for normalization of this dataset. This dataset is chosen to represent a study where there is an imbalance between up- and down-regulated genes, so the current normalization methods are expected to fail.

### Affymetrix spike-in data

The two spike-in data sets were produced by Affymetrix [29] and are part of the assessment procedure at the Affycomp II website [30]. Dataset HGU133A is based on a latin-square experiment with 42 arrays and overall 42 spiked-in genes at various concentrations ranging from 0.0 to 512 pM. Each concentration was performed with three replicates, and each array contains 22,283 probes.

Dataset HGU95A spike-in is one of the data used in the original assessment by [31]. It consists of 20 experiments arranged in a latin-square design, with 14 genes spiked-in at 14 different concentrations ranging from 0.0 to 1024 pM. Each concentration has two replicates, and each array contains 12,626 probes.

## Results

Figure 1 shows the RAplot of the square-root of the array-effect test statistic as a function of the logarithm of the residual standard deviation for the Golden-Spike data, showing the array-to-array variability vs residual variance from the probe-level linear model. Black points correspond to probesets that were spiked in with a nominal fold change ≥ 2, while the black line represents the quantile regression curve using *τ *= 0.6. A total of 8,409 genes lying below this curve were used as the least-variant set of genes for normalization.

Using probe intensities summarized according to the MAS5 algorithm, we performed several normalizations, i.e. global normalization (also known as 'constant normalization'), quantile normalization and invariant-set normalization. Additionally, we performed both a global normalization and a loess normalization using the set of genes with known FC = 1. Since the FC1 genes are the ideal housekeeping genes, these should provide the theoretically best normalization method.

**Figure 2:**
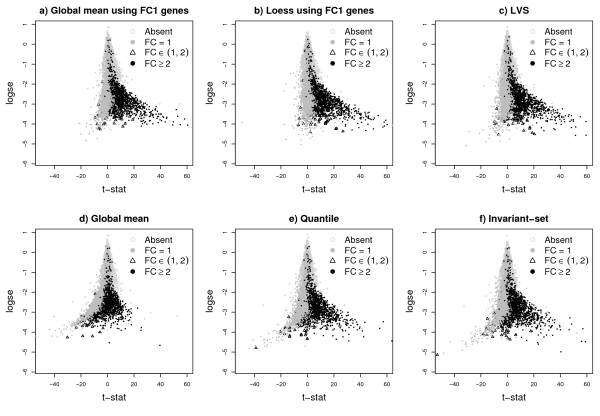
**Plot of the t-statistic versus the log standard-error**. Plot of the t-statistic versus the log standard-error for MAS5 expression values of the Golden-Spike data normalized using different methods. All normalization were performed after summarization of probe intensities. The FC1-based normalizations are ideal, and in real non-spike-in studies are not possible. LVS-normalization is closet to the FC1-based normalization. The others show negative bias for FC1 genes and suppressed values for genes with FC≥ 2.

Figure 2 shows the plots of the regular t-statistics vs the log-standard error of the difference of the group means. All current normalization procedures (global-mean, quantile and invariant-set) introduce a severe bias in the distribution of the t-statistics, where we would expect only over-expressed genes. Instead, the normalization step introduces a set of falsely under-expressed genes (the mass of grey points to the left of the y-axis), mostly coming from FC1 genes. Furthermore, the global-mean normalization, and to some extent the quantile and invariant-set, suppress the expression of genes with high FCs (black points to the right of the y-axis). As seen below, both of these features lead to worse false discovery rates. As expected, the FC1-based normalization methods work well, but of course in real experiments these genes are never known. Finally, LVS normalization produces a t-statistic distribution similar to that obtained using known FC1 genes.

Similar results for RMA expression values (after RMA background correction, and median polish summarization of PM values) are given in Figure S1, Additional file 1.

**Figure 3:**
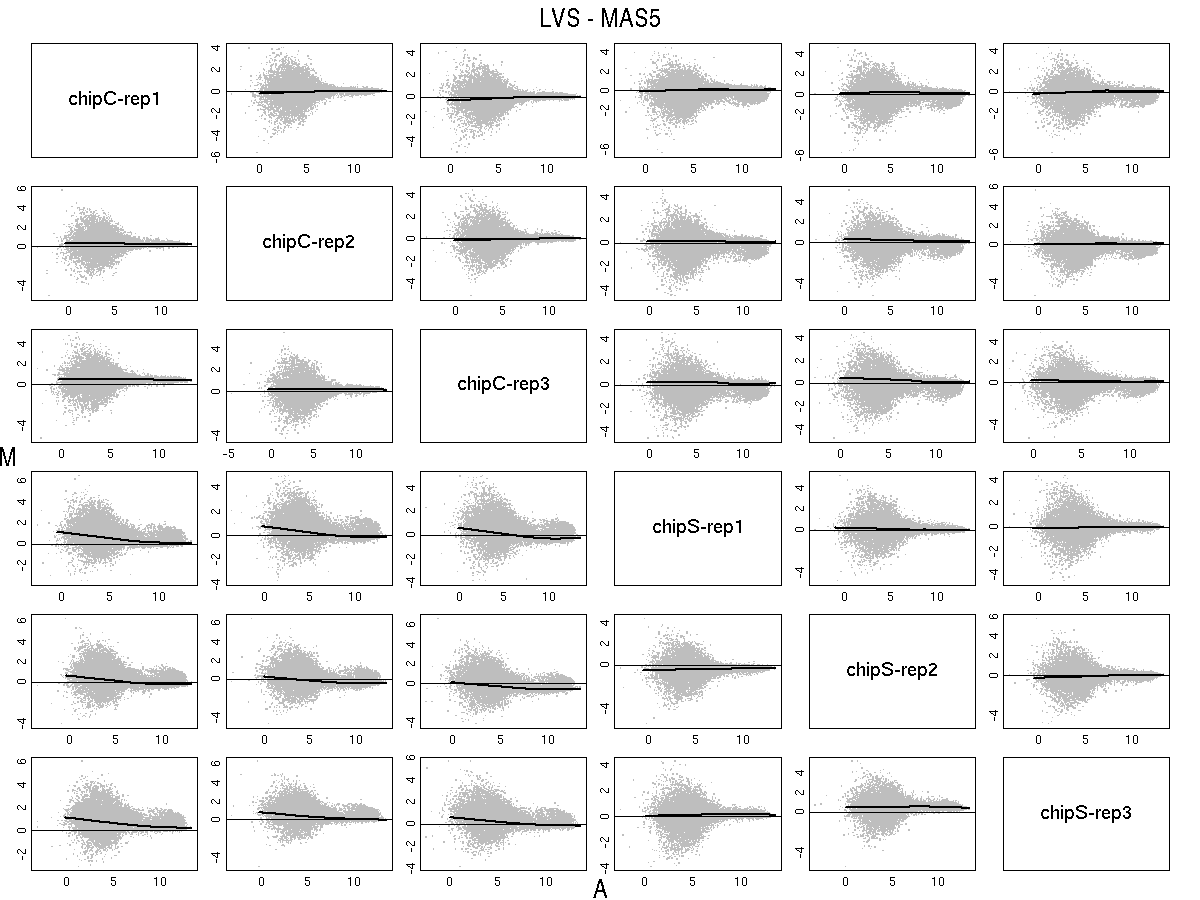
**MA plots**. MA plots of each pair of samples of the Golden-Spike data using MAS5 values (below the diagonal) and after normalization with LVS (above the diagonal). Loess curves, computed from the LVS genes, were drawn in think lines. As expected the normalization has removed any trend.

To show that the LVS normalization procedure had properly removed any trend, we produced paired MA plots (Figure 3) between the array and the pseudo-median array. In each plot we draw a loess curve along the LVS genes.

**Figure 4:**
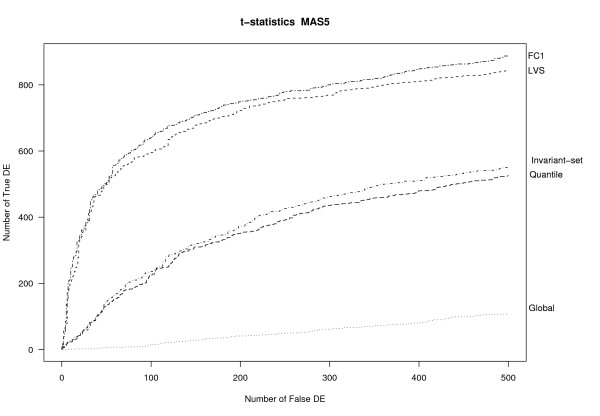
**OC curves**. OC curves for different normalization applied to MAS5-expression values of the Golden-Spike data. FC = 1 refers to the loess normalization on FC1 genes.

¿From a practical point of view, the most important property of a preprocessing algorithm is that it leads to downstream analyses with good operating characteristics (OC). In the downstream analysis of this dataset, the genes are ranked based on the standard t-statistic. Similar results were obtained using a moderated t-statistic instead [32] (see Figure S2, Additional file 1). Figure 4 shows the OC curves for several methods applied to the MAS5 expression data. Curves were drawn up to a maximum of 500 false-positive genes. The LVS normalization performed much better than the quantile, invariant-set or global normalizations, and quite close to the ideal FC1-based normalization. The areas under the curve (AUC) were 0.78 for LVS, 0.057 for global normalization 0.42 for invariant-set, 0.40 for quantile and 0.82 for FC1 normalization; in this computation, the OC curves were standardized to have unit maximum.

### Affymetrix spike-in

The LVS algorithm was evaluated also on the well-known Affymetrix spike-in experiments, namely the HGU-95Av2 and the HGU133A data. The performance of the LVS method was tested within the framework of the affy competition [33,34], using for expression summarization and background correction the methods adopted by the standard MAS5 and RMA algorithms. The reports automatically created by the Affycomp II website [30] are available in the Additional files 2, 3, 4, 5.

**Figure 5:**
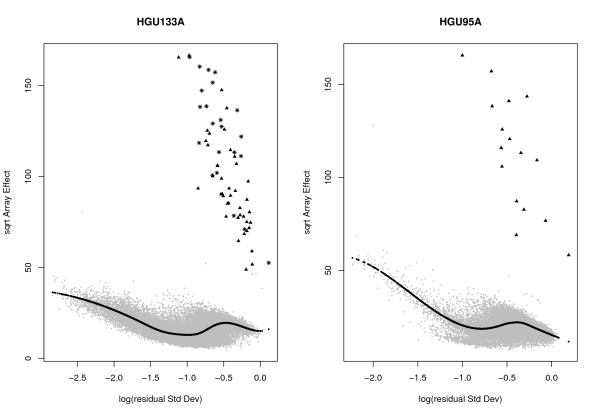
**RA-plots for spike-in data**. RA-plots for both HGU133A and HGU95A spike in data. These plots show the array-to-array variability vs residual variance from the probe-level linear model. The black line represents the fitted values from a quantile regression with *τ *= 0.6. The triangles represent the spiked-in genes. The stars are the new spike-ins according to MCGee *et al*. (2006).

Figure 5 shows the RA-plots of both the HGU133A and the HGU95v2 spike-in experiments. The signal in these datasets is clearly simpler than in the Golden-Spike data (Figure 1), with a clean separation between the spike-in genes (black points) and the mass of unexpressed or nonDE genes (grey points). Illustrating the value of the RAplot, an additional set of 22 genes (black stars) is clearly separated from the mass of non-spike-in probes (for a total of 64 spike-ins); in fact, these correspond to the 22 additional spike-ins found by [35] in this dataset. In our experience, in real data, the pattern in these RAplots is highly unusual; it is produced mainly because there are too few spike-in genes in the experiments.

**Figure 6:**
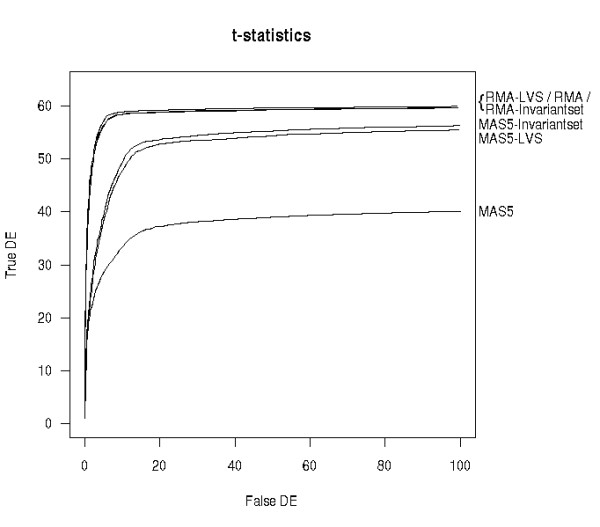
**OC curves for HGU133A spike-in data**. OC curves for different normalizations applied to either MAS5 or RMA expression measures for the HGU133A spike-in experiment. The standard t-statistic was used as the criterion to setup the curve.

Because of the small number of spike-in genes and the clean separation between spiked and non-spiked genes (Figure 5), we do not expect a big difference in performance between LVS and other normalization methods. Figure 6 shows the OC curves for the HGU133A dataset, based on the standard t-statistic and the whole set of 64 spike-ins. For RMA-based expression values, no big difference was observed among normalization procedures (quantile, invariant-set and LVS). However, for MAS5 expression values, a sharp improvement was obtained using the LVS and invariant-set normalization compared to global normalization. Given the limited noise present in the Affymetrix spike-in data, the selection of the subset of genes for normalization is expected to have little impact.

## Discussion and Conclusion

We have presented a new algorithm called LVS for normalizing high-density oligonucleotide arrays based on a set of genes with the least variability across samples. Array-to-array variation is estimated through a robust linear model fitted at the probe level. In noisy and complex datasets such as the Golden-Spike data, LVS normalization outperforms other normalization procedure. In simple spike-in experiments where very few genes are expressed or spiked, all methods of normalization should work equally well. In real experiments, the normalization step does make a difference [2,36], and in this case it is safer to use a more robust method that relies on fewer assumptions.

In the Golden-Spike data it seems clear that the signal observed for unexpressed genes was due to both experimental artifacts and nonspecific hybridizations. The latter occurs because probes associated with unexpressed genes might bind to other mRNA species with high concentration. When these are differentially expressed, the non-specific binding will lead to false discoveries. So, assuming that probes associated with unexpressed genes should be nonDE might indeed be wrong; in recent large arrays, these probes can be expected to be the majority. The complexity of non-specific binding also means that it is important to have more realistic spike-in experiments with a large number of expressed genes.

Any normalization procedure is supposed to equalize the distribution of non-varying genes across samples, thus correcting for any random or non-biological systematic variation. Obviously the determination of the non-varying genes must rely on pre-normalized data. Most current procedures make certain assumptions that would allow one to use the full collection of genes as the reference set, and no analysis is needed to identify them. LVS exploits the information in the probe-level data to determine a pre-specified proportion of least-variant features across samples. In contrast with the invariant-set method, which uses pairwise comparisons between each array and a reference array, LVS uses the full collection of arrays. This partly explains the better performance of the LVS compared to the invariant-set method for the Golden-Spike data.

Because of the intrinsic random noise in microarray experiments, a sufficiently large number of genes should be selected for normalization. Normalization based on a small set of genes, such as the housekeeping-gene or invariant-set normalization, might be ineffective with noisy data. The LVS algorithm allows a reasonably large proportion of genes to be selected as a reference set; we get a stable result over a range of proportions from 40–60%.

One of the key assumptions in current normalization procedures is that there is a balance between up- and down-regulated genes. This explains the failure of the current procedures in the Golden-Spike data. In real studies, can we always expect balanced expression? In lab experiments, e.g. with knock-out mice, an unbalanced proportion of over/under-expressed genes may reasonably occur. Haslett et al. [12], for example, reported a relevant bias towards over-expression in muscle-related genes (135 of the 185 declared DE). A similar unbalanced pattern was reported by [11] and [13].

The problem is that, in practice, the assumptions underlying the normalization procedures are rarely checked, so it is never certain that the data are properly normalized. For example, even for clinical data with balanced expression levels, we showed in [21] that the commonly-used quantile normalization was biased for low-intensity genes. This means that we need a robust and safe procedure that relies on fewer assumptions. We believe that LVS normalization is a step in that direction.

## Authors' contributions

SC performed the analysis, designed the package and wrote the paper. YP performed the analysis and co-wrote the paper. DV developed the package and co-wrote the paper. All authors read and approved the final manuscript.

## Supplementary Material

Additional file 1 Supplementary Report Normalization of oligonucleotide arrays based on the least-variant set of genes. This file contains some supplementary figures. Click here for file

Additional file 2 Bioconductor Expression Assessment Tool for Affymetrix Oligonucleotide Arrays (affycomp). This report presents the automatic assessment of the LVS normalization method, with MAS5-style summarization, based on the Affymetrix HGU 95 spike-in experiment, generated by the Affycomp website [30] Click here for file

Additional file 3 Bioconductor Expression Assessment Tool for Affymetrix Oligonucleotide Arrays (affycomp). This report presents the automatic assessment of the LVS normalization method, with RMA-style summarization, based on the Affymetrix HGU 95 spike-in experiment, generated by the Affycomp website [30] Click here for file

Additional file 4 Bioconductor Expression Assessment Tool for Affymetrix Oligonucleotide Arrays (affycomp). This report presents the automatic assessment of the LVS normalization method, with MAS5-style summarization, based on the Affymetrix HGU 133 spike-in experiment, generated by the Affycomp website [30] Click here for file

Additional file 5 Bioconductor Expression Assessment Tool for Affymetrix Oligonucleotide Arrays (affycomp). This report presents the automatic assessment of the LVS normalization method, with RMA-style summarization, based on the Affymetrix HGU 133 spike-in experiment, generated by the Affycomp website [30] Click here for file
