# Supplementary material for: Normalization of oligonucleotide arrays based on the least-variant set of genes
Source: BMC Bioinformatics. 2008 Mar 5;9:140. doi: 10.1186/1471-2105-9-140 (PMC2324100; doi:10.1186/1471-2105-9-140)
Supplement: Additional file 1 — Supplementary Report Normalization of oligonucleotide arrays based on the least-variant set of genes. This file contains some supplementary figures. [file 1471-2105-9-140-S1.pdf]

Supplementary Report: Normalization of oligonucleotide arrays based on the least-variant set of genes

Stefano Calza et al.

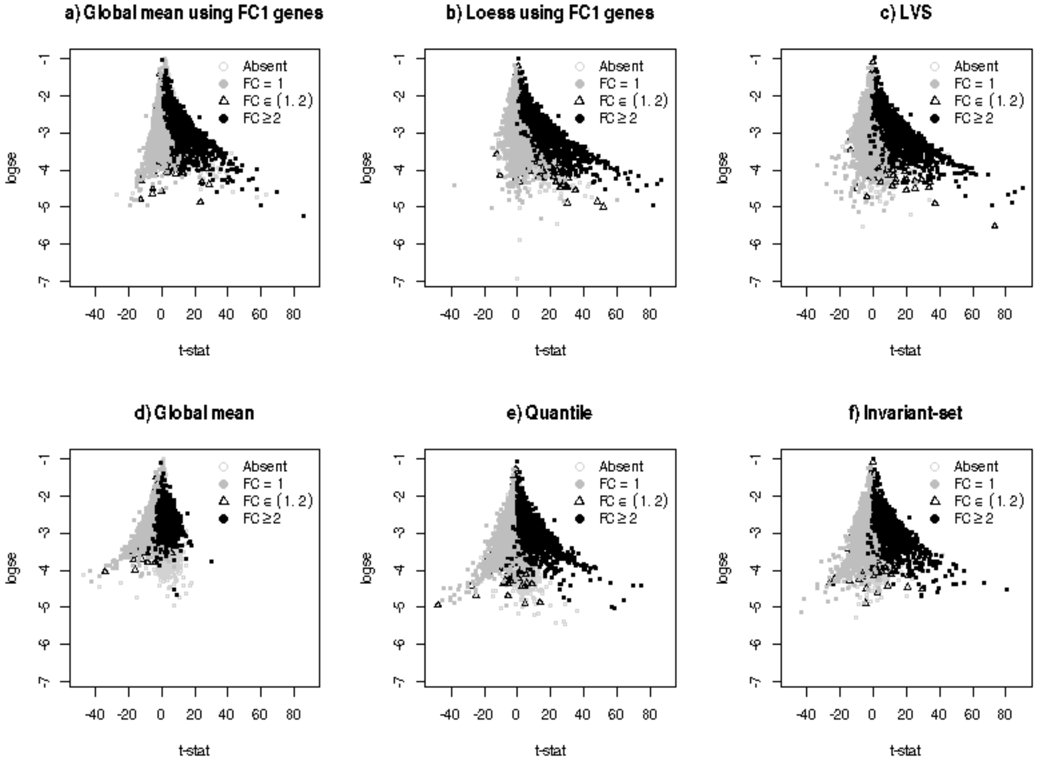

Figure S1: Plot of the t-statistic versus the log standard-error for **RMA expression values** of the Golden-Spike data normalized using different methods. All normalization were performed after summarization of probe intensities. The FC1-based normalizations are ideal, and in real non-spike-in studies are not possible. LVS-normalization is closest to the FC1-based normalization. The others show negative bias for FC1 genes and suppressed values for genes with FC≥ 2.

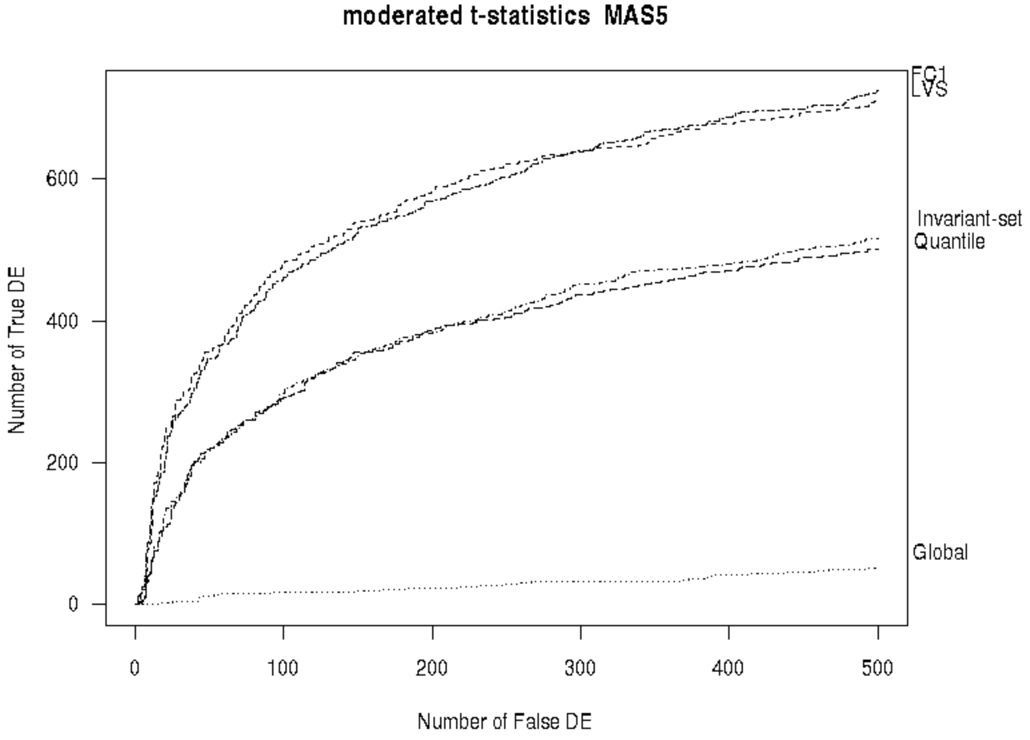

Figure S2: OC curves for different normalizations applied to MAS5 expression values of the Golden-Spike data using **the moderated t-statistic**. 'FC=1' refers to the loess normalization on FC1 genes.

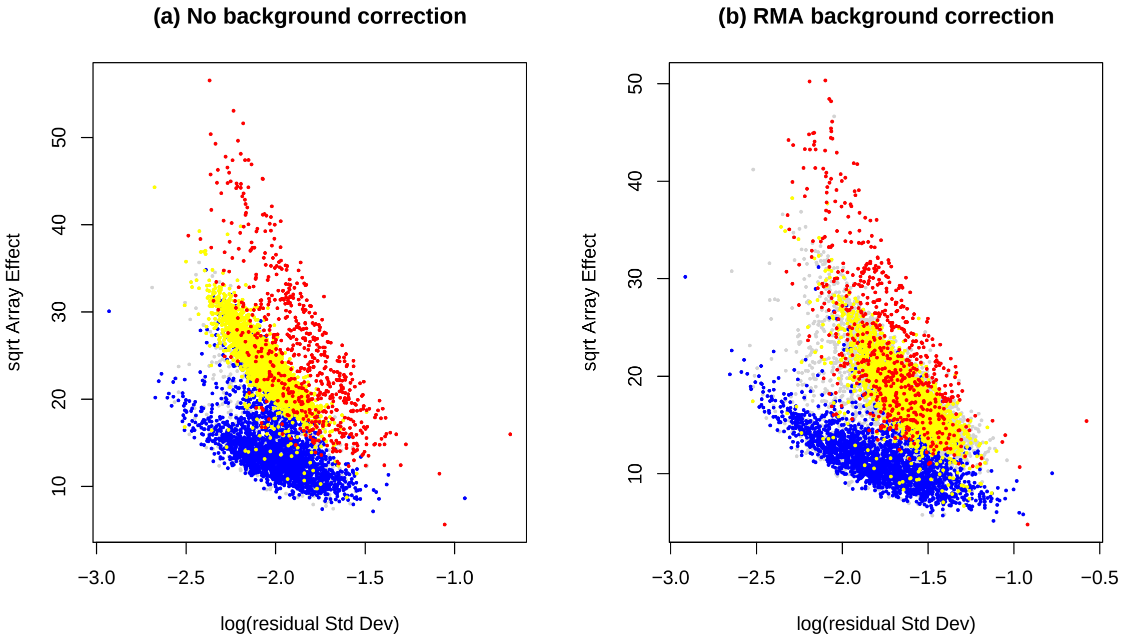

Figure S3: Residual Std Dev (log scale) vs Array Effect (sqrt scale) plots. (a) No background correction and (b) RMA background correction. Yellow points are non-present genes, blue points are FC1 genes and red points are genes with FC>2
