# Supplementary material for: Normalization of oligonucleotide arrays based on the least-variant set of genes
Source: BMC Bioinformatics. 2008 Mar 5;9:140. doi: 10.1186/1471-2105-9-140 (PMC2324100; doi:10.1186/1471-2105-9-140)
Supplement: Additional file 2 — Bioconductor Expression Assessment Tool for Affymetrix Oligonucleotide Arrays (affycomp). This report presents the automatic assessment of the LVS normalization method, with MAS5-style summarization, based on the Affymetrix HGU 95 spike-in experiment, generated by the Affycomp website [30] [file 1471-2105-9-140-S2.pdf]

Bioconductor Expression Assessment Tool for Affymetrix Oligonucleotide Arrays (affycomp)

April 30, 2007

Contents

In this report only assessment using the **HGU95** spike-In experiment are presetned. Figures 2,3, and 4b are therefore ommited.

```
fullname: Stefano Calza - Yudi Pawitan
method: LVS-MAS5
nickname: LVS
competition: NO

Overall signal to ratio assessment:

      slope        R2  medianSD Null FC IQR Null FC 99.9% Rank
LVS 0.70074 0.8603064 0.6073669   0.8448566      4.496629 2133

Expected Rank (out of 12626) for genes with fold change of 2
when all other genes are not differentially expressed
stratified by nominal concentration.

           LVS
0.25:0    1376
0.5:0.25  1725
1:0.5     2659
2:1       2116
4:2       1954
8:4       1823
16:8      1877
32:16     1853
64:32     1958
128:64    2236
```

256:128	2549
512:256	3635
1024:512	3889

Table 1 for Spike-in Data:

	LVS
Signal detect slope	7.049972e-01
Signal detect R2	8.635739e-01
AUC (FP<10)	2.253270e-01
AUC (FP<15)	2.449507e-01
AUC (FP<25)	2.726380e-01
AUC (FP<100)	3.424215e-01
AFP, call if fc>2	3.049531e+03
ATP, call if fc>2	1.284362e+01
IQR	2.898288e+00
Obs-intended-fc slope	6.936238e-01
Obs-(low)int-fc slope	6.426354e-01
FC=2, AUC (FP<10)	6.412338e-02
FC=2, AUC (FP<15)	6.431362e-02
FC=2, AUC (FP<25)	6.507555e-02
FC=2, AUC (FP<100)	6.669908e-02
FC=2, AFP, call if fc>2	3.039964e+03
FC=2, ATP, call if fc>2	3.714286e+00

Table 2 for Spike-in Data:

	LVS
null log-fc IQR	0.84485664
null log-fc 99%	3.58021906
null log-fc 99.9%	4.49662852
low AUC	0.05742574
med AUC	0.00000000
high AUC	0.00000000
weighted avg AUC	0.04306931
25% SD	0.29512430
Median SD	0.60736694
75% SD	0.96611776
99% SD	1.56391680
low.slope	0.70672645
med.slope	0.80937228
high.slope	0.45756770
low.R2	0.15995373

med.R2	0.54273012
high.R2	0.41181401
0.25:0	1.09267552
0.5:0.25	0.88032989
1:0.5	0.53312302
2:1	0.70792897
4:2	0.77215197
8:4	0.83107842
16:8	0.80696888
32:16	0.81763848
64:32	0.77074834
128:64	0.66258450
256:128	0.56380922
512:256	0.32824309
1024:512	0.28717553

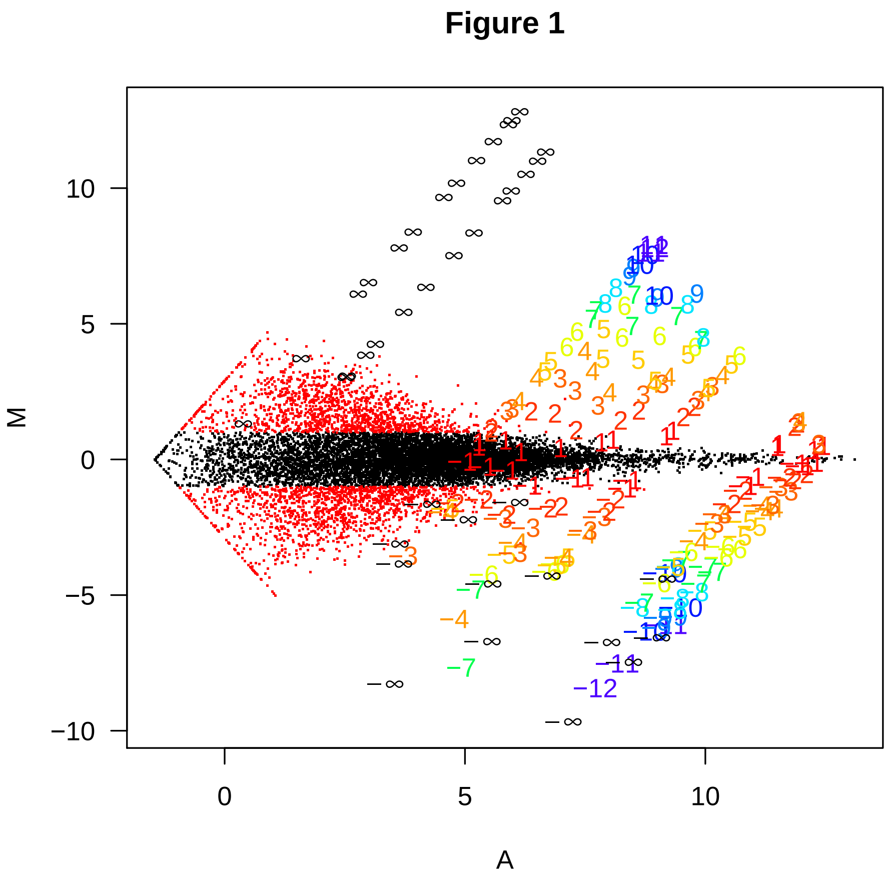

Figure 1: The MA plot shows log fold change as a function of mean log expression level. A set of 14 arrays representing a single experiment from the Affymetrix spike-in data are used for this plot. A total of 13 sets of fold changes are generated by comparing the first array in the set to each of the others. Spiked-in genes are symbolized by numbers representing the nominal $\log_2$ fold change for the gene. Non-differentially expressed genes with observed fold changes larger than 2 are plotted in red. All other probesets are represented with black dots.

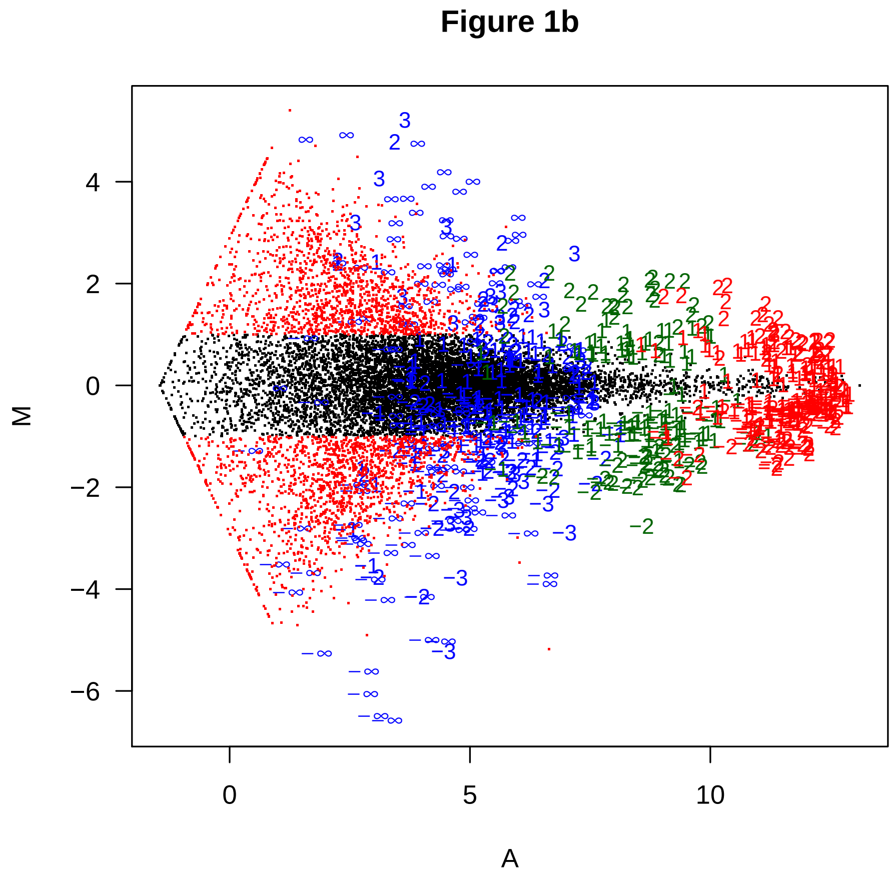

Figure 1b: The MA plot shows log fold change as a function of mean log expression level. A set of 28 arrays representing a single experiment from the Affymetrix spike-in data are used for this plot. Fold changes are generated for all possible comparisons of the the first 14 arrays and the second 14 arrays. Spiked-in genes are symbolized by numbers representing the nominal $\log_2$ fold change for the gene. Of the genes that are spiked to be differentially expressed, only genes with small nominal fold changes are shown. The colors represent four different groups: nominal concentration of genes being compared less than or equal to 2 picoMolar (blue), between 4 and 32 picoMolar (green), greater than or equal to 64 picoMolar (blue). Non-differentially expressed genes with observed fold changes larger than 2 are plotted in red. All other probesets are represented with black dots.

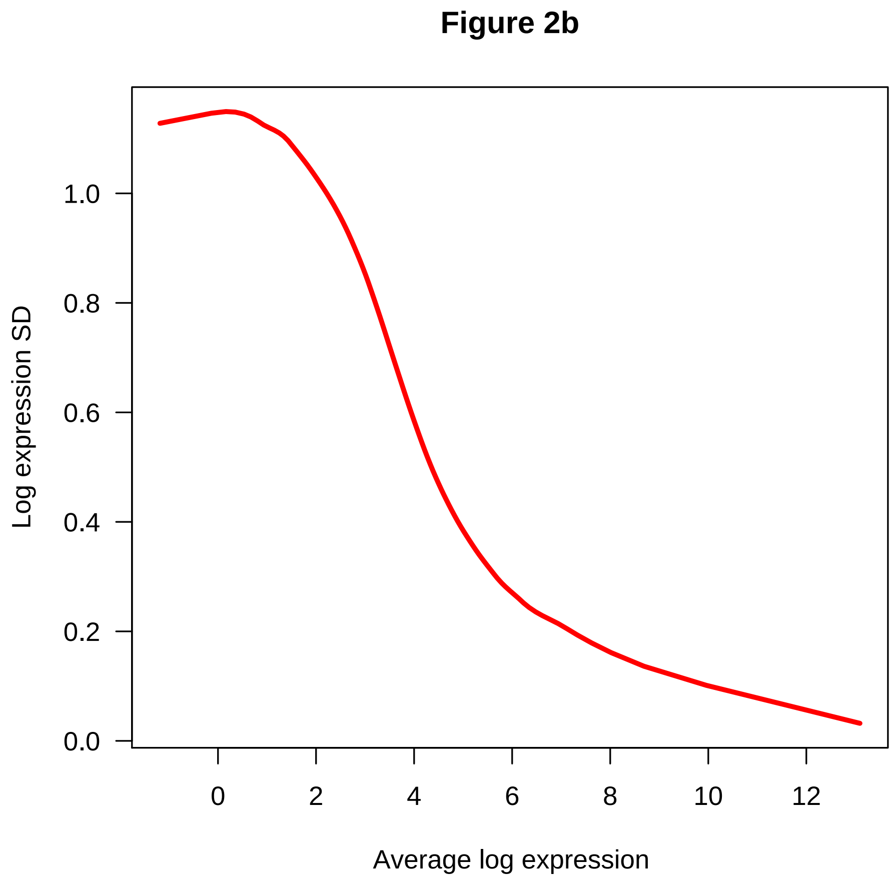

Figure 2b: For each non-spiked-in gene in the 28 arrays used in Figure 1b, we calculate the mean log expression and the observed standard deviation across the 28 replicates. The resulting scatterplot is smoothed to generate a single curve representing mean standard deviation as a function of mean log expression.

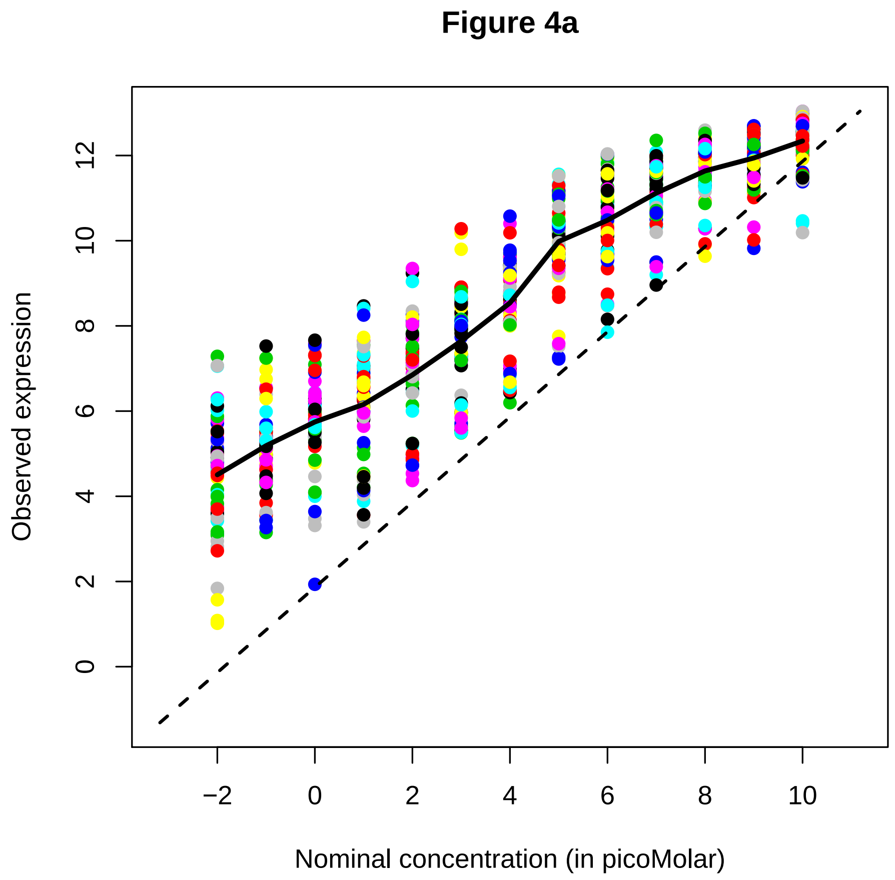

Figure 4a) Average observed log_2 intensity plotted against nominal log_2 concentration for each spiked-in gene for all arrays in Affymetrix spike-In experiment. The dashed line has the ideal slope of 1.

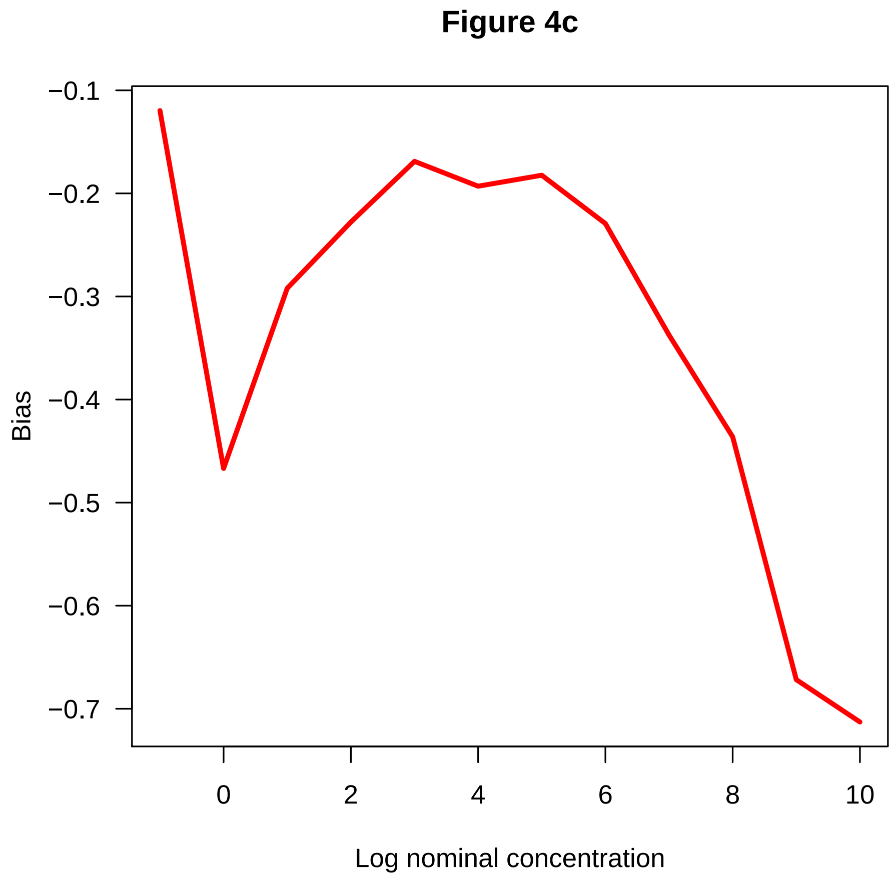

Figure 4c) Using the 28 arrays of Figure 1b, we compute local slopes. As the slopes shown in Figure 4a), the local slopes represent the expected observed log fold-change for probesets with true fold-change of 2 but they are presented as a function of the total nominal probeset concentration in the two samples being compared. In theory the local slopes should be one so we show the bias (difference between the observed local slope and one).

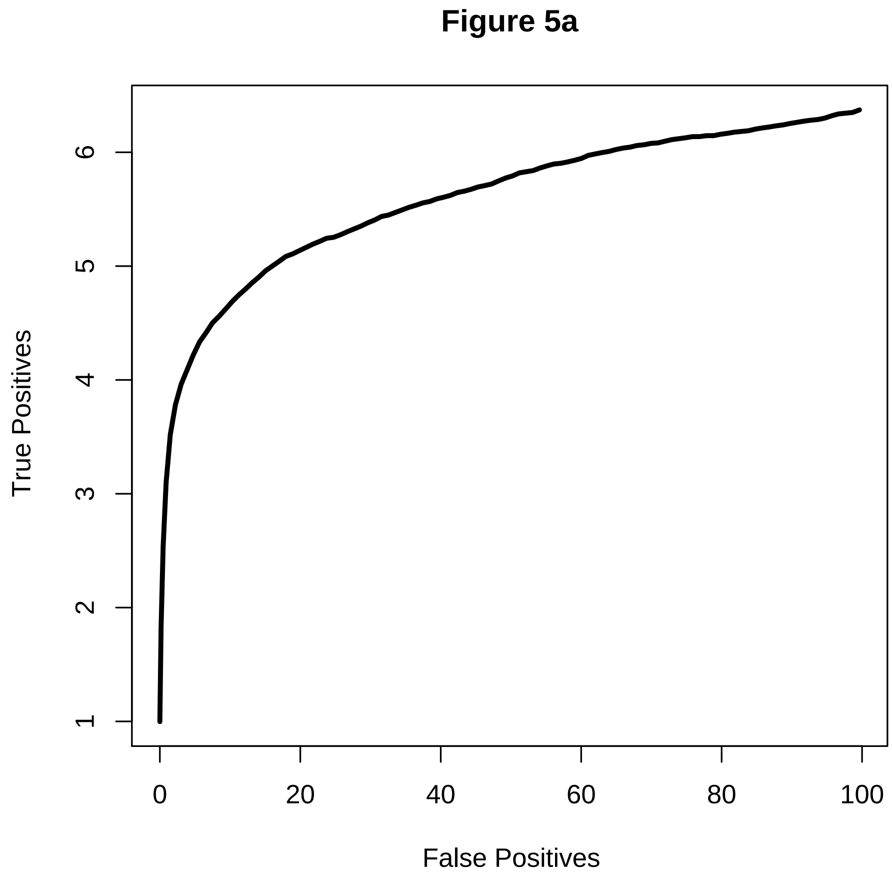

Figure 5a) A typical identification rule for differential expression filters genes with fold change exceeding a given threshold. This figure shows average ROC curves which offer a graphical representation of both specificity and sensitivity for such a detection rule. Average ROC curves based on comparisons with nominal fold changes ranging from 2 to 4096.

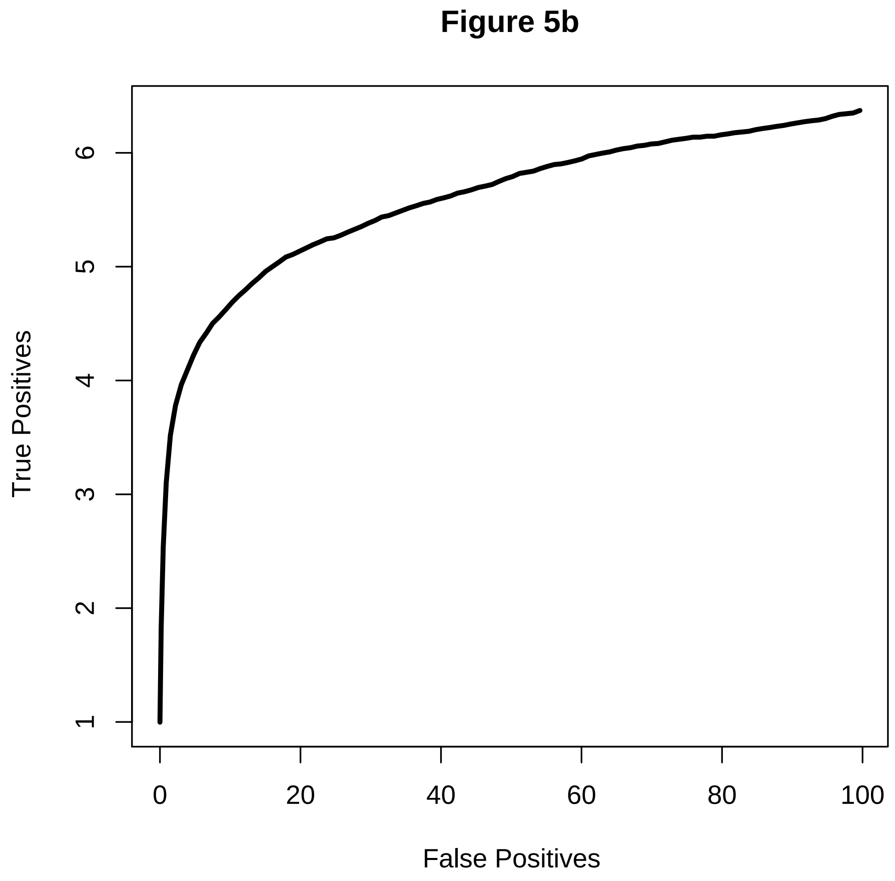

Figure 5b) As 5a) but with nominal fold changes equal to 2.

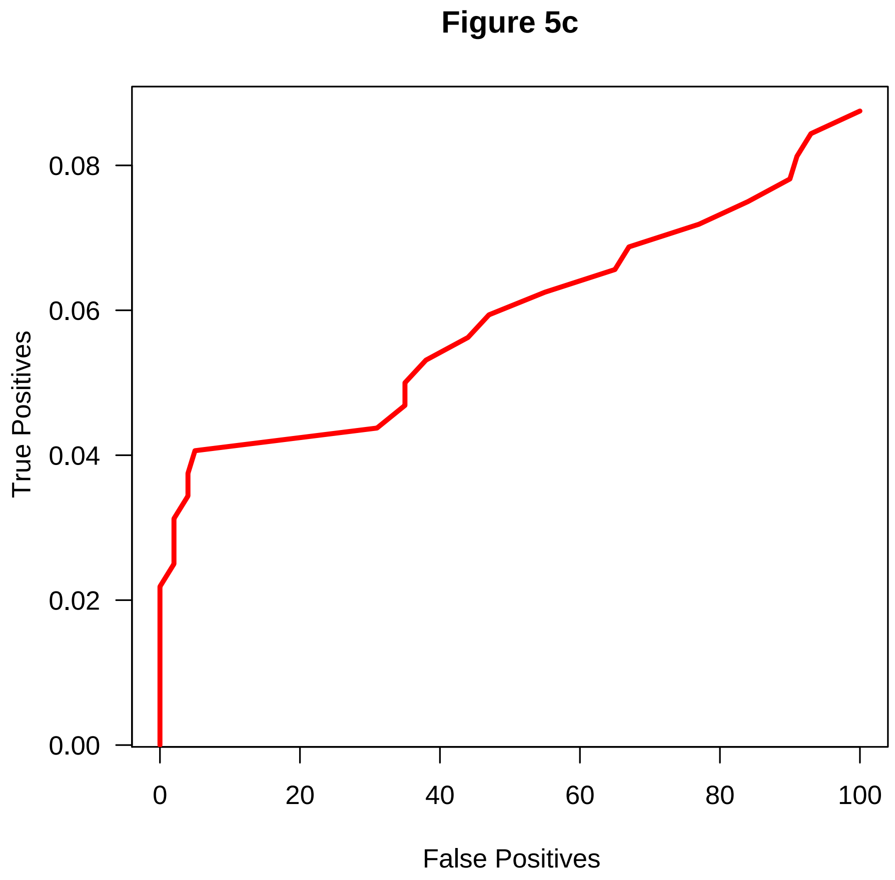

Figure 5c) As 5a) but for comparisons with both nominal concentrations less than or equal to 2 picoMolar.

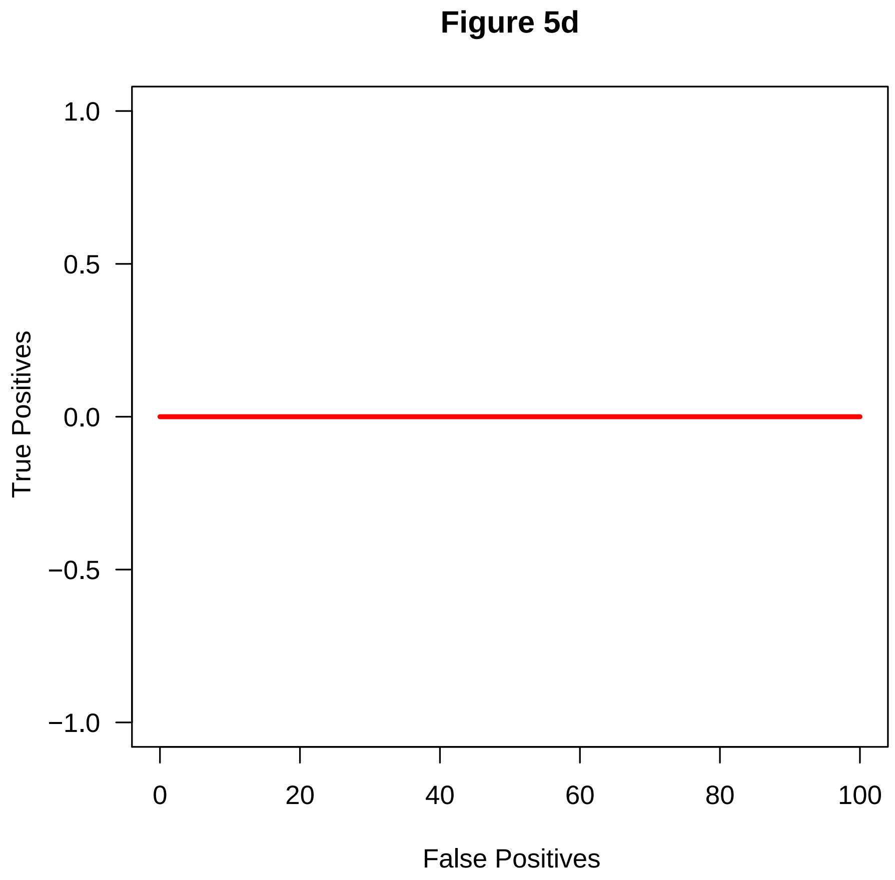

Figure 5d) As 5a) but for comparisons with both nominal concentrations between 4 and 32 picoMolar and nominal fold changes less or equal to 4.

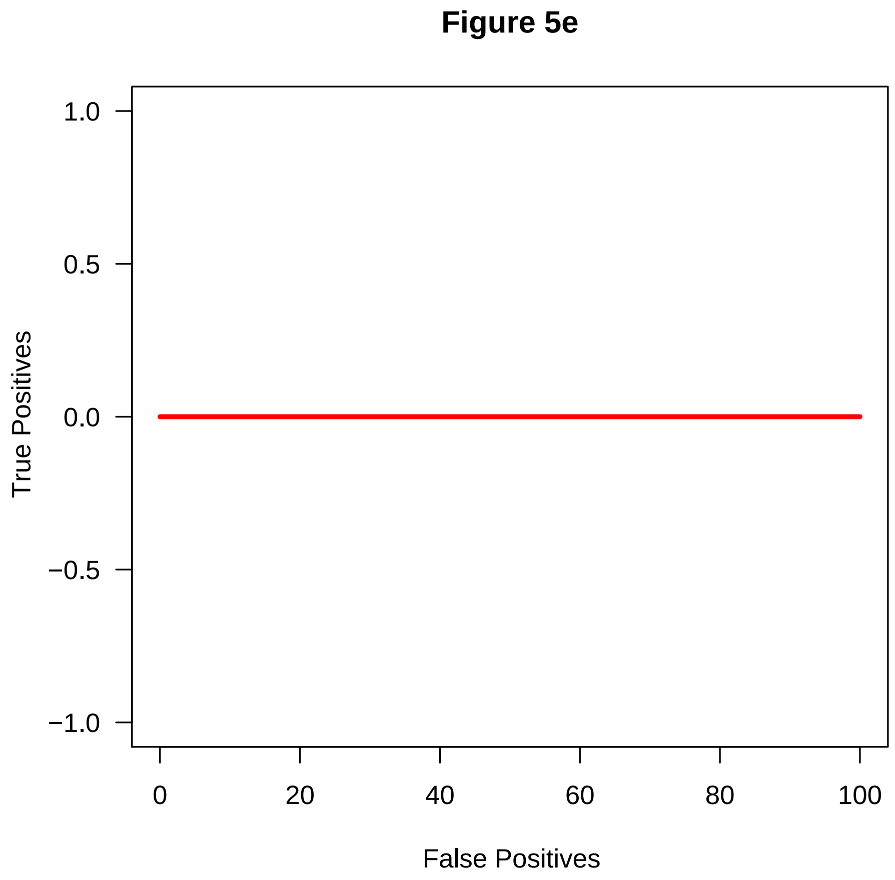

Figure 5e) As 5a) but for comparisons with both nominal concentrations larger or equal to 64 and with nominal fold changes less than or equal to 4.

Figure 6a

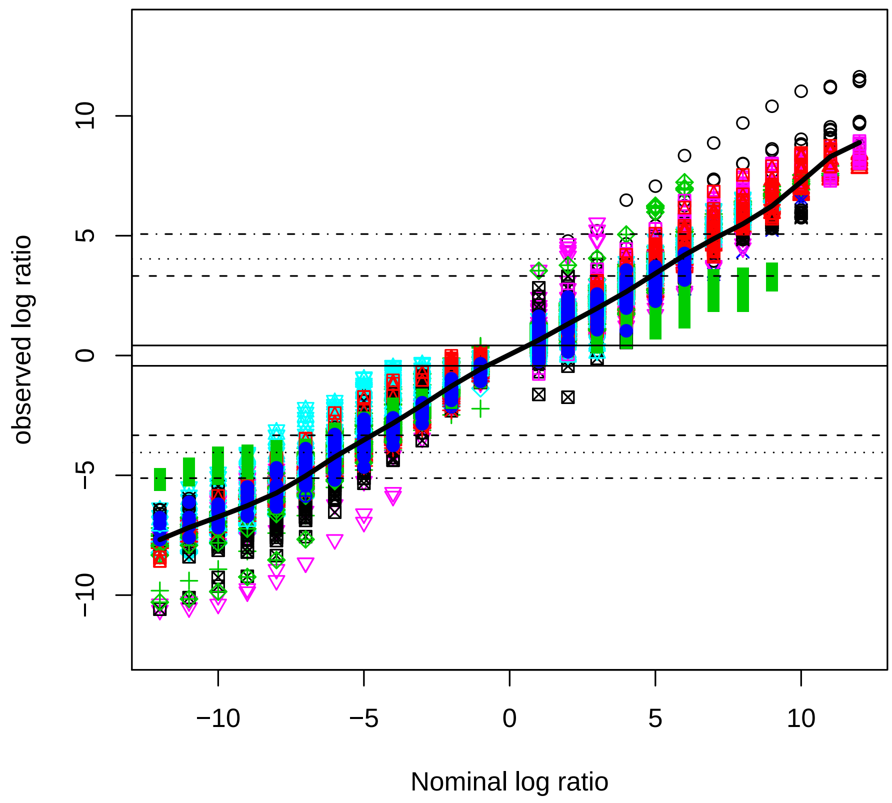

Figure 6a) Observed log fold changes plotted against nominal log fold changes. The dashed lines represent highest, 25th highest, 100th highest, 25th percentile, 75th percentile, smallest 100th, smallest 25th, and smallest log fold change for the genes that were not differentially expressed.

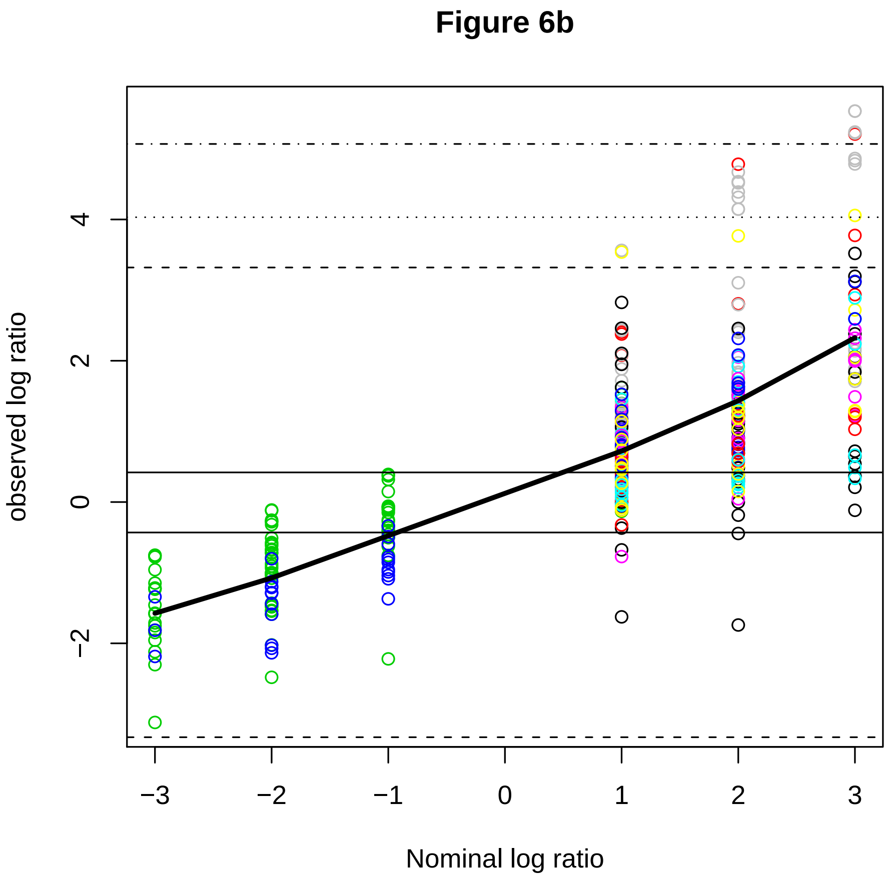

Figure 6b) Like a) but the observed fold changes were calculated for spiked in genes with nominal concentrations no higher than 2pM.
