# Supplementary material for: Normalization of oligonucleotide arrays based on the least-variant set of genes
Source: BMC Bioinformatics. 2008 Mar 5;9:140. doi: 10.1186/1471-2105-9-140 (PMC2324100; doi:10.1186/1471-2105-9-140)
Supplement: Additional file 5 — Bioconductor Expression Assessment Tool for Affymetrix Oligonucleotide Arrays (affycomp). This report presents the automatic assessment of the LVS normalization method, with RMA-style summarization, based on the Affymetrix HGU 133 spike-in experiment, generated by the Affycomp website [30] [file 1471-2105-9-140-S5.pdf]

Bioconductor Expression Assessment Tool for Affymetrix Oligonucleotide Arrays (affycomp)

April 27, 2007

Contents

In this report only assessment using the **HGU133** spike-In experiment are presetned. Figures 2,3, and 4b are therefore ommited.

```
fullname: Stefano
method: LVS-RMA
nickname: LVS
competition: NO

Overall signal to ratio assessment:

        slope       R2   medianSD Null FC IQR Null FC 99.9% Rank
LVS 0.6792494 0.897898 0.08141693   0.1485833     0.4490963    1

Expected Rank (out of 12626) for genes with fold change of 2
when all other genes are not differentially expressed
stratified by nominal concentration.

             LVS
0.125:0      877
0.25:0.125  2541
0.5:0.25    1517
1:0.5         10
2:1            2
4:2            2
8:4            2
16:8           2
32:16          2
64:32          2
```

128:64	2
256:128	2
512:256	3

Table 1 for Spike-in Data:

	LVS
Signal detect slope	0.6792494
Signal detect R2	0.8978981
AUC (FP<10)	0.5248487
AUC (FP<15)	0.5607029
AUC (FP<25)	0.6157672
AUC (FP<100)	0.7790727
AFP, call if fc>2	1.7252747
ATP, call if fc>2	32.9157509
IQR	0.2742211
Obs-intended-fc slope	0.6778150
Obs-(low)int-fc slope	0.3039009
FC=2, AUC (FP<10)	0.3891953
FC=2, AUC (FP<15)	0.4206889
FC=2, AUC (FP<25)	0.4718906
FC=2, AUC (FP<100)	0.6118001
FC=2, AFP, call if fc>2	0.2380952
FC=2, ATP, call if fc>2	10.9047619

Table 2 for Spike-in Data:

	LVS
null log-fc IQR	0.14858327
null log-fc 99%	0.33240626
null log-fc 99.9%	0.44909632
low AUC	0.53115931
med AUC	0.87895647
high AUC	0.92905862
weighted avg AUC	0.61860962
25% SD	0.06485936
Median SD	0.08141693
75% SD	0.09726067
99% SD	0.16139629
low.slope	0.20577620
med.slope	0.71055639
high.slope	0.79575252
low.R2	0.07455979

med.R2	0.63840539
high.R2	0.65854883
0.125:0	0.16917235
0.25:0.125	0.08916387
0.5:0.25	0.13073485
1:0.5	0.42244366
2:1	0.64928547
4:2	0.73330417
8:4	0.66883006
16:8	0.66109559
32:16	0.82458931
64:32	0.94763151
128:64	1.01554316
256:128	0.80930857
512:256	0.55788716

Figure 1

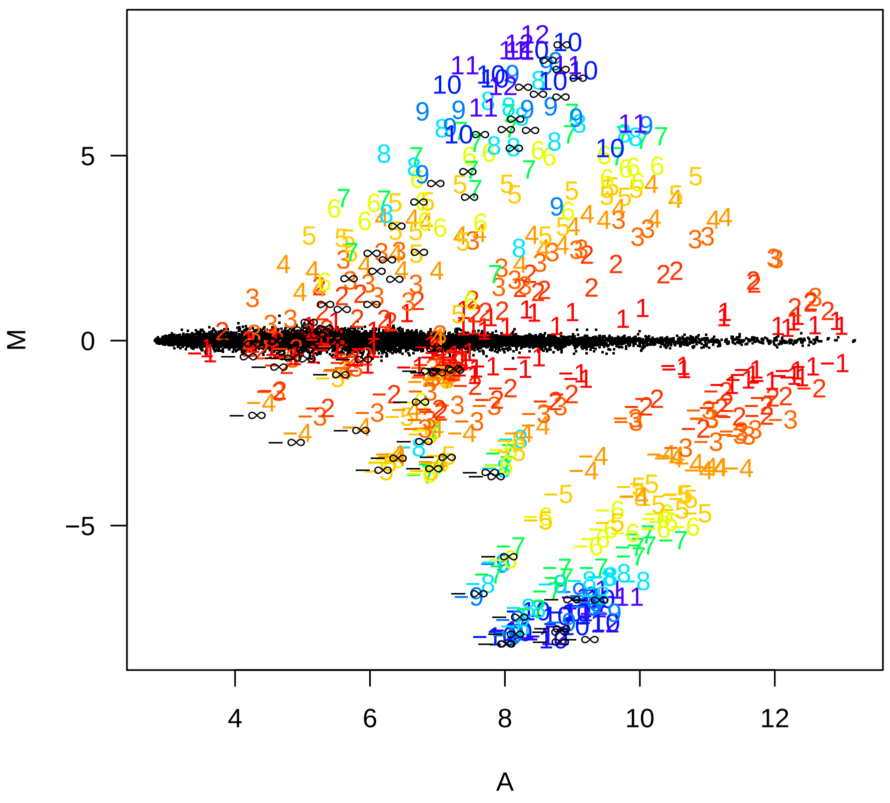

Figure 1: The MA plot shows log fold change as a function of mean log expression level. A set of 14 arrays representing a single experiment from the Affymetrix spike-in data are used for this plot. A total of 13 sets of fold changes are generated by comparing the first array in the set to each of the others. Spiked-in genes are symbolized by numbers representing the nominal $\log_2$ fold change for the gene. Non-differentially expressed genes with observed fold changes larger than 2 are plotted in red. All other probesets are represented with black dots.

Figure 1b

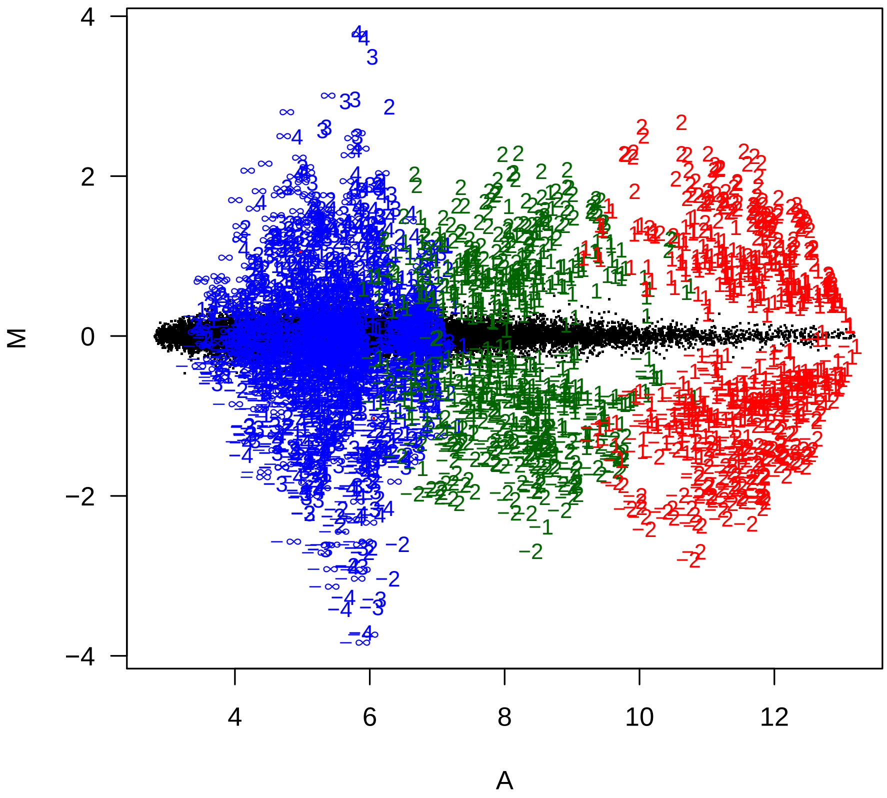

Figure 1b: The MA plot shows log fold change as a function of mean log expression level. A set of 28 arrays representing a single experiment from the Affymetrix spike-in data are used for this plot. Fold changes are generated for all possible comparisons of the the first 14 arrays and the second 14 arrays. Spiked-in genes are symbolized by numbers representing the nominal $\log_2$ fold change for the gene. Of the genes that are spiked to be differentially expressed, only genes with small nominal fold changes are shown. The colors represent four different groups: nominal concentration of genes being compared less than or equal to 2 picoMolar (blue), between 4 and 32 picoMolar (green), greater than or equal to 64 picoMolar (blue). Non-differentially expressed genes with observed fold changes larger than 2 are plotted in red. All other probesets are represented with black dots.

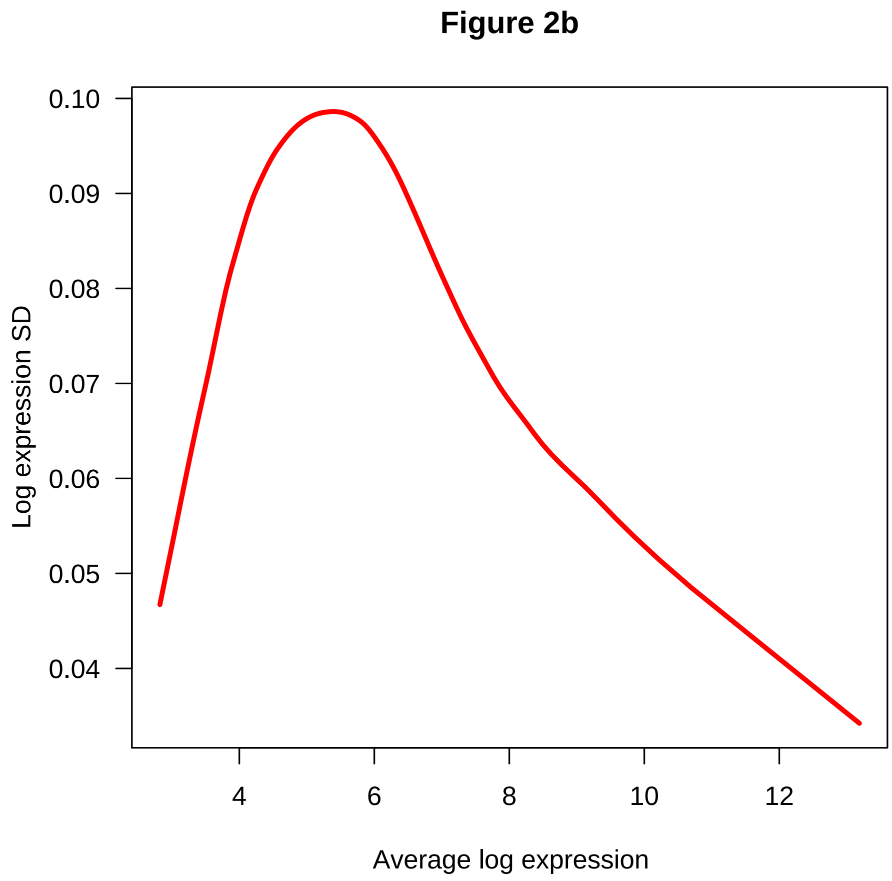

Figure 2b: For each non-spiked-in gene in the 28 arrays used in Figure 1b, we calculate the mean log expression and the observed standard deviation across the 28 replicates. The resulting scatterplot is smoothed to generate a single curve representing mean standard deviation as a function of mean log expression.

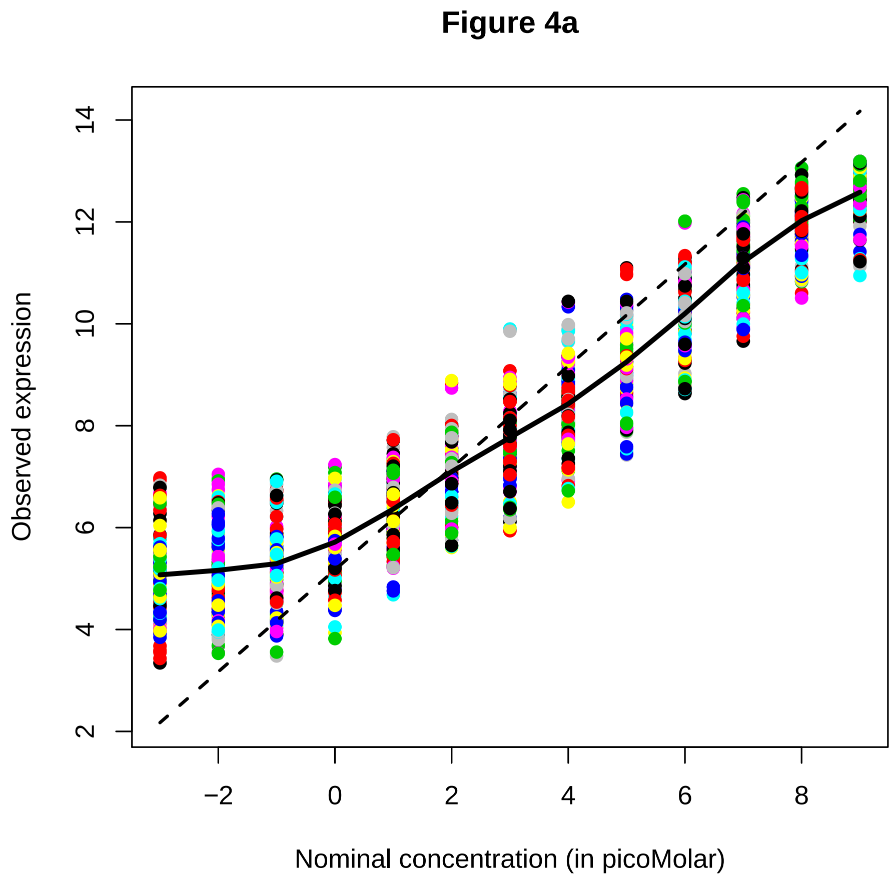

Figure 4a) Average observed log_2 intensity plotted against nominal log_2 concentration for each spiked-in gene for all arrays in Affymetrix spike-In experiment. The dashed line has the ideal slope of 1.

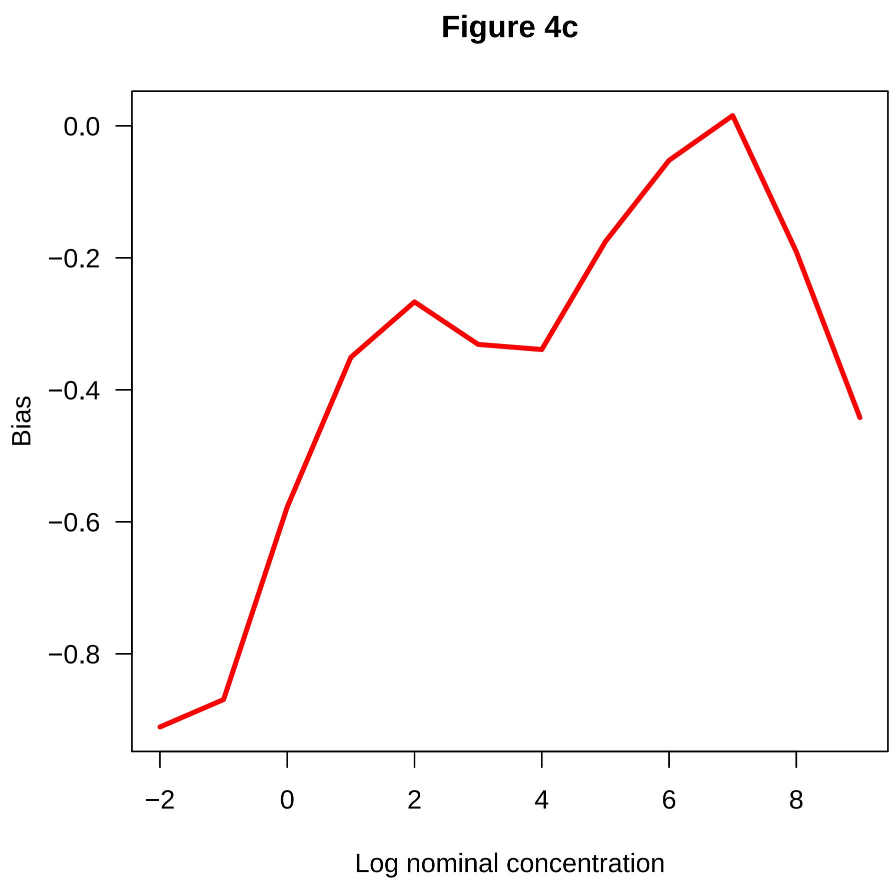

Figure 4c) Using the 28 arrays of Figure 1b, we compute local slopes. As the slopes shown in Figure 4a), the local slopes represent the expected observed log fold-change for probesets with true fold-change of 2 but they are presented as a function of the total nominal probeset concentration in the two samples being compared. In theory the local slopes should be one so we show the bias (difference between the observed local slope and one).

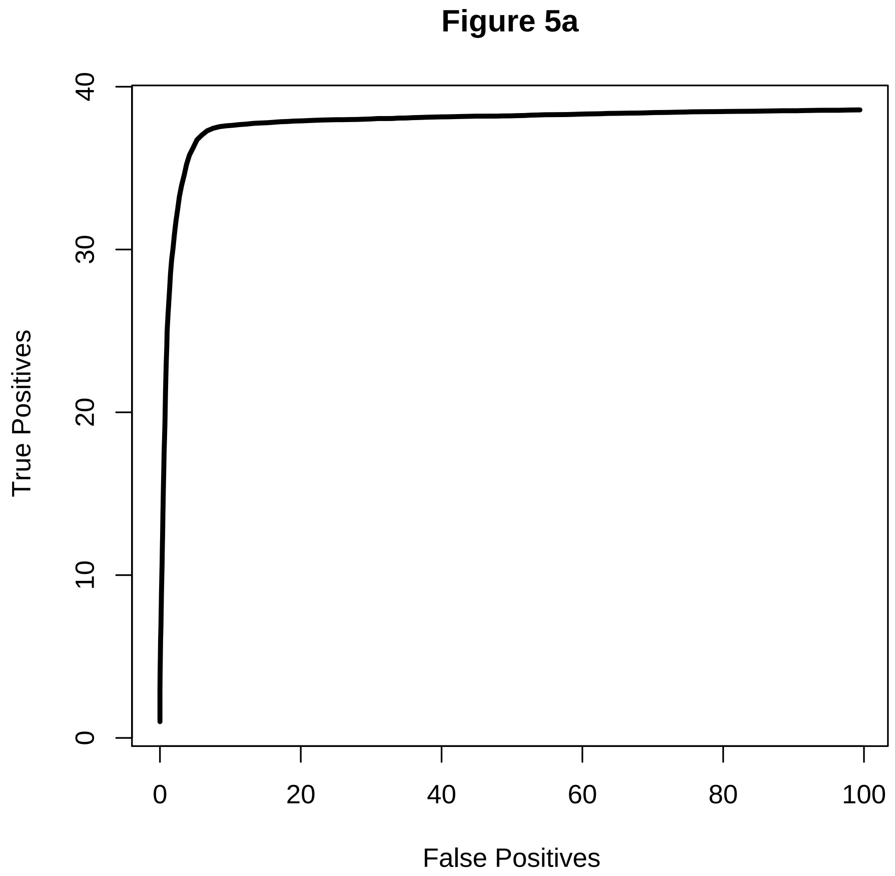

Figure 5a) A typical identification rule for differential expression filters genes with fold change exceeding a given threshold. This figure shows average ROC curves which offer a graphical representation of both specificity and sensitivity for such a detection rule. Average ROC curves based on comparisons with nominal fold changes ranging from 2 to 4096.

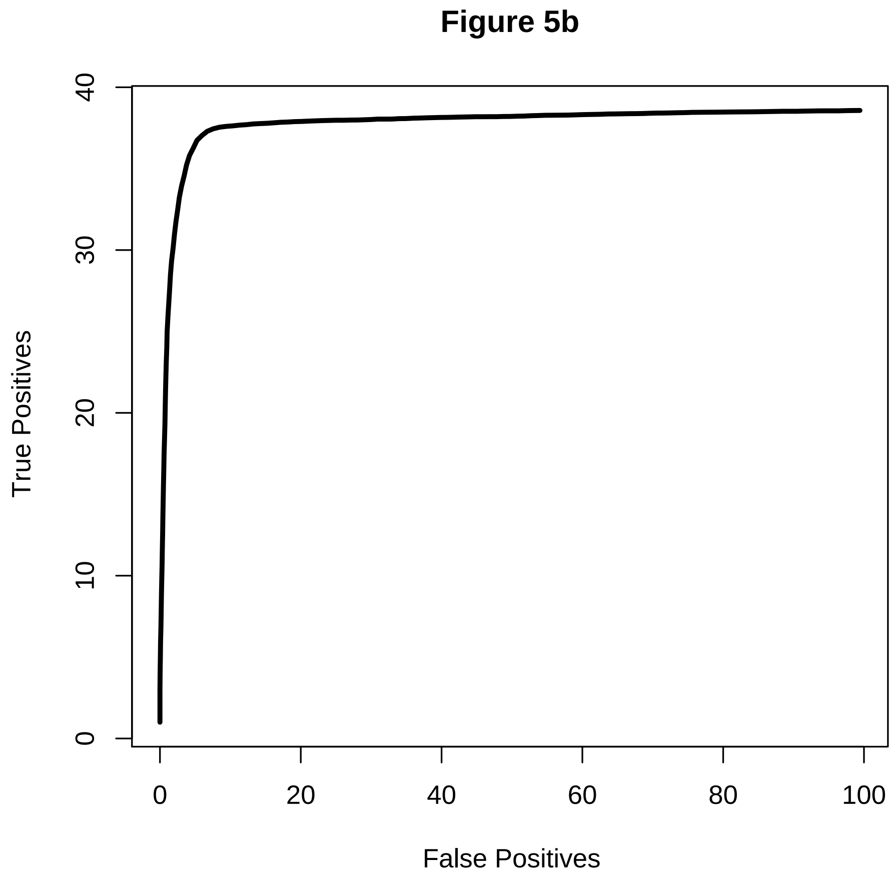

Figure 5b) As 5a) but with nominal fold changes equal to 2.

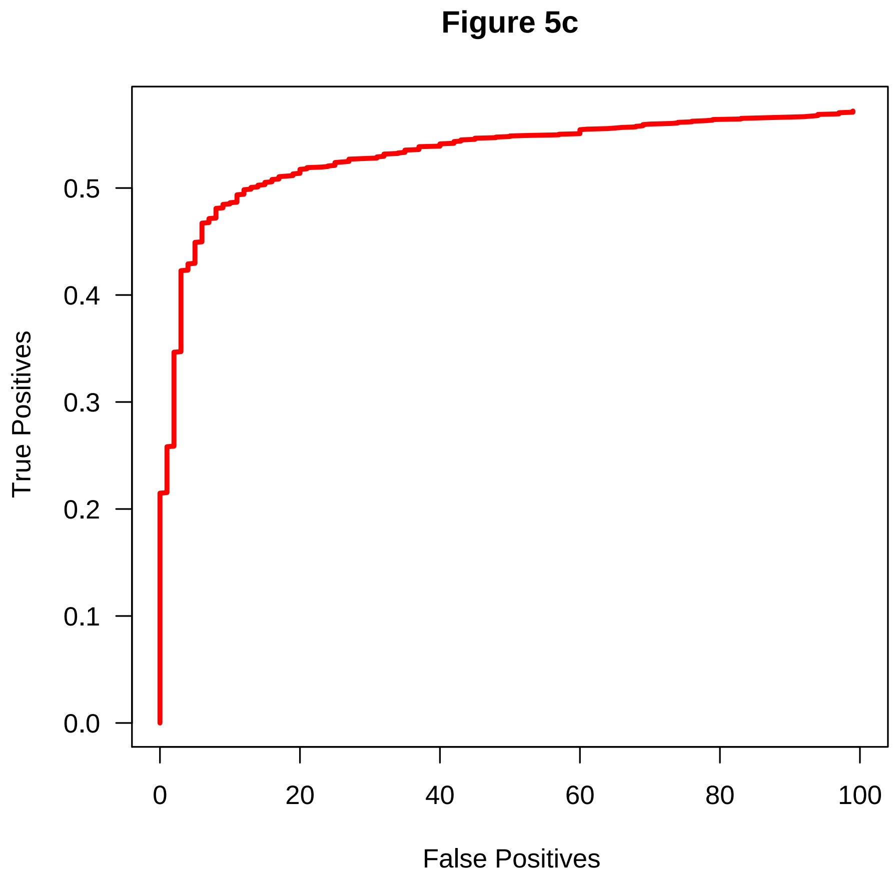

Figure 5c) As 5a) but for comparisons with both nominal concentrations less than or equal to 2 picoMolar.

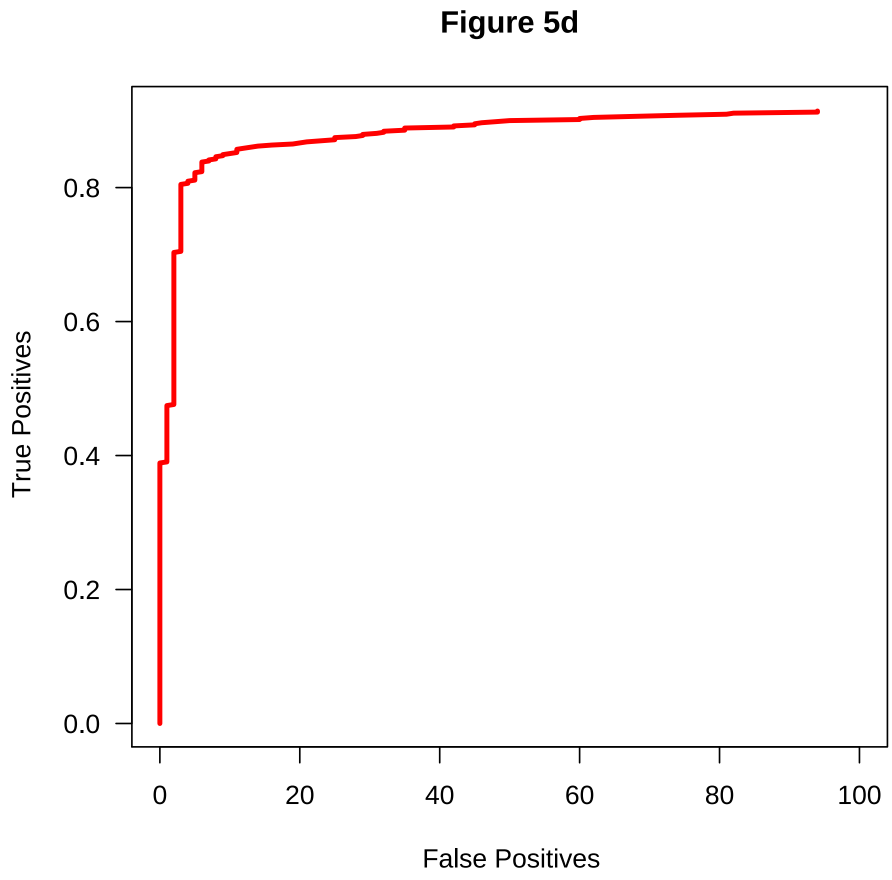

Figure 5d) As 5a) but for comparisons with both nominal concentrations between 4 and 32 picoMolar and nominal fold changes less or equal to 4.

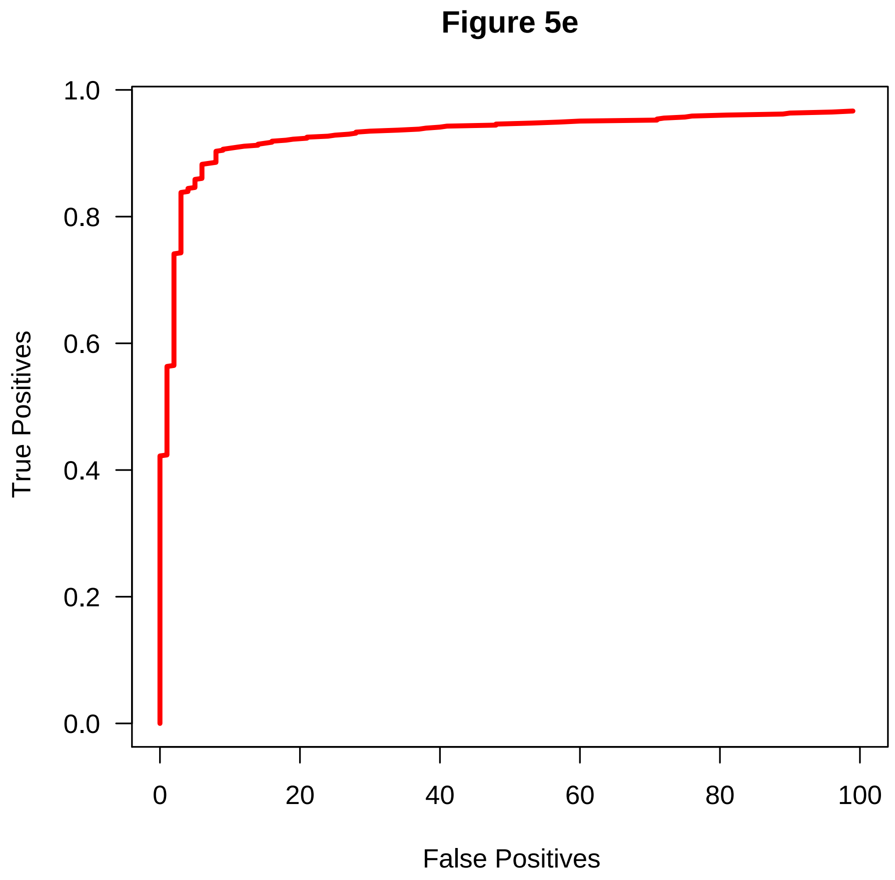

Figure 5e) As 5a) but for comparisons with both nominal concentrations larger or equal to 64 and with nominal fold changes less than or equal to 4.

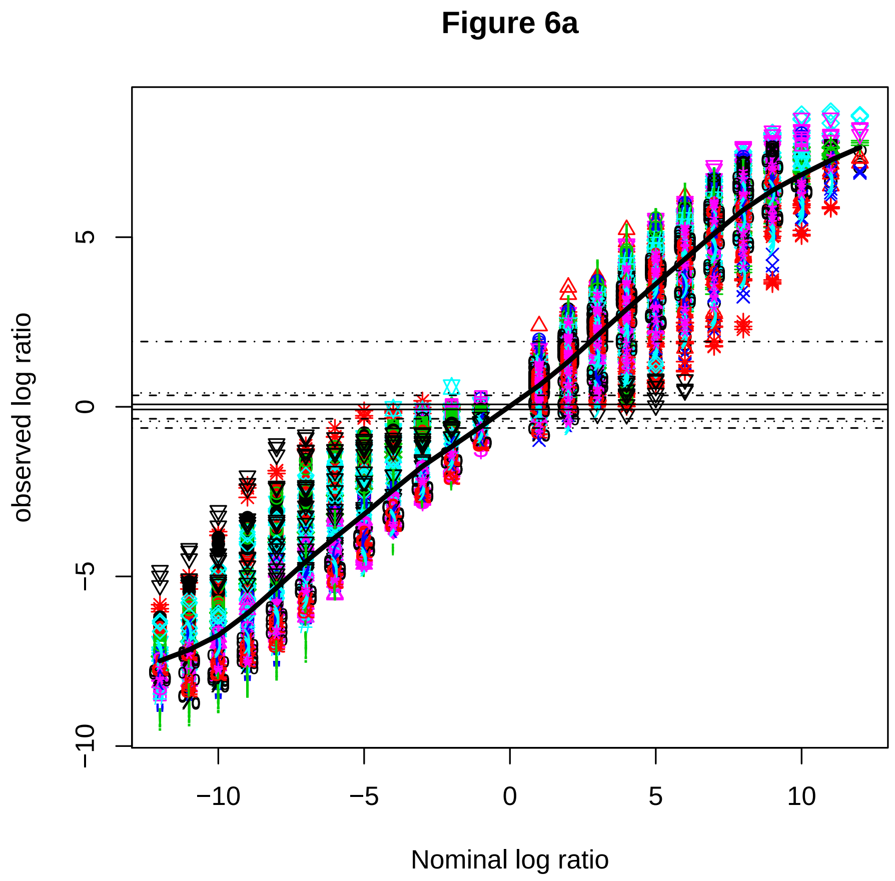

Figure 6a) Observed log fold changes plotted against nominal log fold changes. The dashed lines represent highest, 25th highest, 100th highest, 25th percentile, 75th percentile, smallest 100th, smallest 25th, and smallest log fold change for the genes that were not differentially expressed.

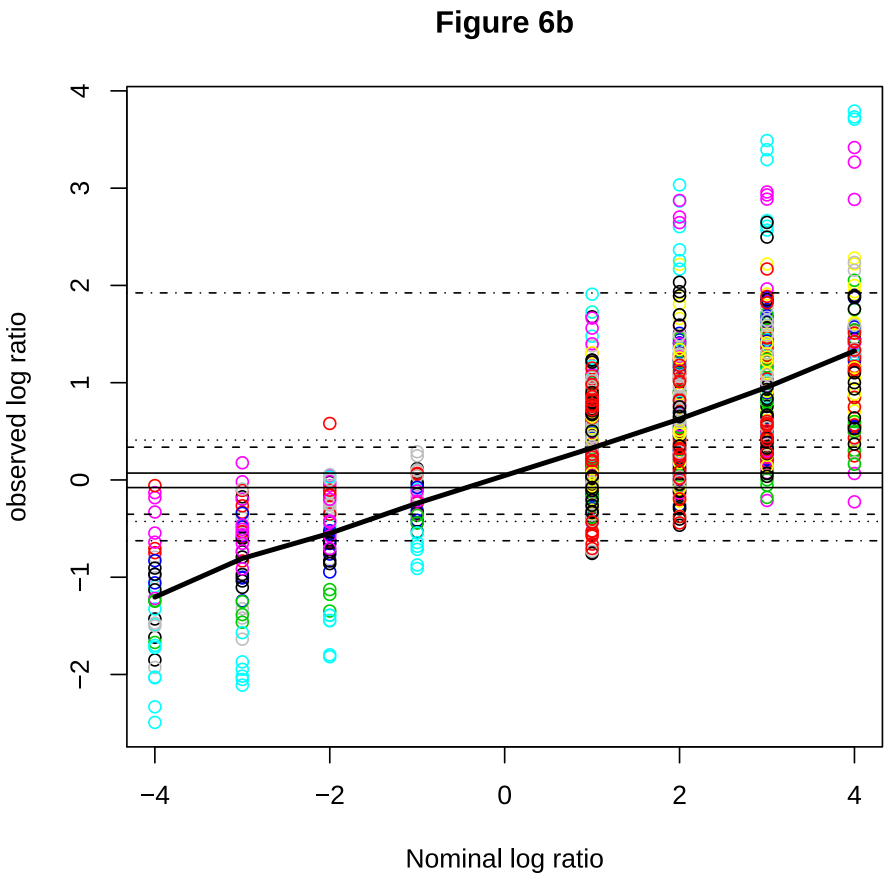

Figure 6b) Like a) but the observed fold changes were calculated for spiked in genes with nominal concentrations no higher than 2pM.
